# Geographical variation in cardiovascular incidence: results from the British Women's Heart and Health Study

**DOI:** 10.1186/1471-2458-10-696

**Published:** 2010-11-15

**Authors:** Lois G Kim, Claire Carson, Debbie A Lawlor, Shah Ebrahim

**Affiliations:** 1 Department of Medical Statistics Unit, Faculty of Epidemiology and Population Health, London School of Hygiene and Tropical Medicine, Keppel St, London. WC1E 7HT, UK; 2 National Perinatal Epidemiology Unit, University of Oxford, Old Road Campus, Headington, Oxford. OX3 7LF, UK; 3 MRC Centre for Causal Analyses in Translational Epidemiology, School of Social and Community Medicine, University of Bristol, Oakfield House, Oakfield Grove, Clifton, Bristol. BS8 2BN, UK; 4 Department of Non-communicable Disease Epidemiology, Faculty of Epidemiology and Population Health, London School of Hygiene and Tropical Medicine, Keppel St, London. WC1E 7HT, UK

## Abstract

**Background:**

Prevalence of cardiovascular disease (CVD) in women shows regional variations not explained by common risk factors. Analysis of CVD incidence will provide insight into whether there is further divergence between regions with increasing age.

**Methods:**

Seven-year follow-up data on 2685 women aged 59-80 (mean 69) at baseline from 23 towns in the UK were available from the British Women's Heart and Health Study. Time to fatal or non-fatal CVD was analyzed using Cox regression with adjustment for risk factors, using multiple imputation for missing values.

**Results:**

Compared to South England, CVD incidence is similar in North England (HR 1.05 (95% CI 0.84, 1.31)) and Scotland (0.93 (0.68, 1.27)), but lower in Midlands/Wales (0.85 (0.64, 1.12)). Event severity influenced regional variation, with South England showing lower fatal incident CVD than other regions, but higher non-fatal incident CVD. Kaplan-Meier plots suggested that regional divergence in CVD occurred before baseline (before mean baseline age of 69).

**Conclusions:**

In women, regional differences in CVD early in adult life do not further diverge in later life. This may be due to regional differences in early detection, survivorship of women entering the study, or event severity. Targeting health care resources for CVD by geographic variation may not be appropriate for older age-groups.

## Background

Geographical variations in coronary heart disease (CHD) and stroke (together termed cardiovascular disease (CVD) here) have been identified and reported for a range of countries in terms of both prevalence [1-16] and incidence [17-21]. Furthermore, a number of studies have investigated the relationship between geographical variation in these outcomes and known risk factors [11,17,18,22-24]. The British Regional Heart Study (BRHS) reported that the north-south differences in CVD incidence in men could largely be explained by classical risk factors (smoking, physical activity, body mass index (BMI), alcohol consumption, systolic blood pressure, serum total cholesterol, occupational social class, and height) [18]. In women, differences in CVD prevalence across four broad regions of the UK (Scotland, North England, Midlands/Wales, and South England) were reported at the baseline survey of the British Women's Heart and Health Study (BWHHS) [11]. The highest prevalence of CVD was observed in Scotland, and the lowest in South England. In contrast to findings in men drawn from the same geographic areas, this variation by region remained after adjustment for known risk factors (age, systolic blood pressure, diastolic blood pressure, total cholesterol, high density lipoprotein cholesterol (HDLc), smoking physical activity, fruit consumption, social class, and use of aspirin/statins). The work presented here extends this to consider geographical variations in the incidence of CVD in women in the BWHHS, using data from seven years of follow-up of the cohort.

## Methods

**Figure 1:**
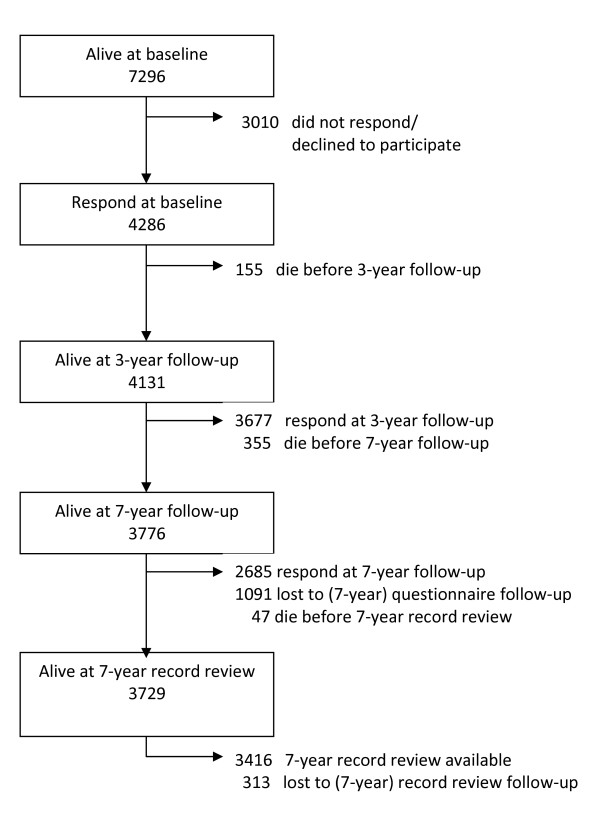
**Flow chart showing response to questionnaires and 7-year record review**.

Methods for the BWHHS have been published previously [11], and were based on the BRHS for men [25]. In summary, one GP practice in each of 23 towns in the UK was selected for the study, matching those towns in the BRHS. Women registered at these practices were invited to complete a baseline questionnaire about health and lifestyle (Additional file 1), and to attend a clinical examination to obtain measurements and blood samples. Details on diagnosed CVD, treatments and risk factors were obtained through nurse-led interview. Of the 7296 women invited between April 1999 and March 2001, 4286 (59%) responded at baseline. A further two questionnaires have since been completed, in 2003 (three-year follow-up; Additional files 2, 3) and 2007 (seven-year follow-up; Additional file 4), with 3677 (86% of baseline responders, 89% of those still alive) and 2685 (63% of baseline responders, 71% of those still alive) responding respectively. Figure 1 shows the flow of the participants through the study. GP practice records were reviewed in tandem with the questionnaires, and all women are flagged with the UK National Health Service Central Register, which provides mortality data via the Office of National Statistics.

Multi-centre (London Multi-centre Regional Ethics Committee) and local research ethics committees provided approval for the study and informed consent was obtained from the women to complete the measurements used in this study, abstract information from medical records, link to the National Health Service Central Register and store data.

### Outcomes

Cardiovascular disease (CVD) was defined as any of angina, unstable angina, myocardial infarction or stroke. Prevalent events were informed by either self-report at baseline or medical record review events dated prior to baseline. Incident events were informed by either self-report at the three- or seven-year follow-up, medical record review, or death certificate, using a cut-off of 30^th ^September 2007 for all sources. CVD deaths were indicated by ICD10 codes I200-259, I516, I600-679, I690-699, G460-469, and G450-3 (underlying or otherwise). Self-reported strokes were only included where symptoms were >24 hours (to exclude potential cases of transient ischaemic attack (TIA) mis-reported as stroke). For potential events identified by review of medical records, a number of criteria were applied in order to count an event as incident CVD for analysis. In doubtful cases a telephone discussion was held with the woman's general practitioner to confirm or reject CVD diagnoses. For MI, at least one of the following was required: (1) ECG evidence of MI, (2) raised cardiac enzyme (including troponin), (3) hospital letter confirming MI. For stroke, at least one of the following was required: (1) report of ischaemic/haemorrhagic stroke on CT or MRI scan, (2) hospital letter confirming stroke, (3) final diagnosis from GP notes is ischaemic/haemorrhagic/subarachnoid/other stroke (in absence of scan and letter). For unstable angina a hospital letter confirming the diagnosis was required.

Dates of CVD events were required for time-to-event analysis. Exact dates were available for events ascertained by record review or death certificates. In the case of events only ascertained by self-report (n = 67, 13% of all events), only a year was given; 31st December was therefore used for the purposes of analysis (those with missing year are excluded (n = 47 where no other incident event occurred)). For prevalent events, the date of the earliest pre-baseline event was used for analysis. Similarly for incident events, the date of the earliest post-baseline event was used.

### Risk factors

In addition to age, data on a variety of known and potential risk factors for CVD were collected at baseline. Smoking status (current, ex or never smoker), family history of CVD (father, mother, brother or sister had a heart attack or stroke), alcohol intake (never, socially, or most days), limited fruit intake (both winter and summer fruit intake less than once a week/never), limited physical activity (never take regular exercise), socioeconomic position, and CVD medications (aspirin (British National Formulary code [26] 02.09), statins and other cholesterol-lowering drugs (code 02.12), beta-blockers (code 02.04), ACE inhibitors (code 02.02.05), and blood-pressure lowering drugs (codes 02.02.01, 02.02.08, 02.05.01-06, 02.06.02, 02.04)) were obtained through the questionnaire. Life-course socioeconomic position (SEP) was calculated as a score out of ten responses, with a high score representing greater deprivation [27]. Responses given as "don't know" to the individual components of the score are treated as missing. However, where the husband's social class was missing or not applicable, the responder's own social class was used where available (available for 392/816 (48%)).

Systolic blood pressure (SBP), waist circumference, obesity (body mass index >30 kg/m^2^) and leg length were obtained through the nurse examination. The presence of diabetes at baseline was identified from self-report (n = 31), record review (n = 19), both self-report and record review (n = 182), fasting glucose higher than 7 mmol/l only (n = 184), or diabetic medicines only (n = 1)). Blood samples were taken after a minimum eight hour fast and were used to obtain fasting glucose, total cholesterol, HDLc and triglycerides[11]. Low density lipoprotein cholesterol (LDLc) was calculated from total cholesterol, HDLc and triglycerides using Friedwald's equation [28]. The Carstairs index [29] of area deprivation (derived from the 1991 census, using data for Great Britain) was calculated using postcodes at baseline [30].

The 23 towns in the BWHHS were separated into four geographical regions used previously [11]: Scotland (comprising Ayr, Dumfermline, Falkirk), North England (comprising Burnley, Carlisle, Darlington, Grimsby, Harrogate, Hartlepool, Scunthorpe, Southport, Wigan), Midlands/Wales (comprising Mansfield, Merthyr Tydfil, Newcastle Under Lyme, Shrewsbury), and South England (comprising Bedford, Bristol, Exeter, Gloucester, Guildford, Ipswich, Lowestoft).

### Statistical methods

Regional differences in both fatal and non-fatal CVD incidence were analysed using Cox regression adjusted for age. Individuals were censored on the date of emigration, date of death, or 30^th ^September 2007. Loss to follow-up for non-fatal incident CVD following non-response to questionnaires (1091/3776 (29%) of those alive at seven-year follow-up) or non-return of record review (313/3730 (8%) of those alive at final record review) is not considered here. However, loss of information regarding non-fatal events is expected to be low since loss to follow-up for both sources is small (n = 180, 5% of those alive at seven-year follow-up).

Further adjustment was made for known and potential risk factors (see Table 1, section A). Corresponding Kaplan-Meier survival functions were also produced for both incident CVD and prevalent/incident CVD combined.

**Table 1:** Baseline characteristics by region

	South England	Midlands/ Wales	North England	Scotland	p-value^^
Number of towns	7	4	9	3	
Contacted	2216	1199	2873	1008	
Responders at baseline (% of invited)	1319 (60%)	770 (64%)	1650 (57%)	547 (54%)	< 0.0005
Responders in 2003 (% baseline responders^§^)	1141 (87%)	633 (82%)	1439 (87%)	464 (85%)	0.09
Responders in 2007 (% baseline responders^§^)	846 (72%)	450 (68%)	1037 (71%)	352 (74%)	0.2

***Classical and potential risk factors for CVD (baseline)***					
Age (mean (SD))	69.3 (5.5)	69.5 (5.6)	69.6 (5.5)	68.9 (5.3)	0.06
Smoking status					< 0.0005
Never	817 (62%)	418 (54%)	864 (52%)	272 (50%)	
Ex	389 (30%)	244 (32%)	584 (36%)	184 (34%)	
Current	108 (8%)	101 (13%)	195 (12%)	91 (17%)	
Missing	5 (0.4%)	7 (0.9%)	7 (0.4%)	0 (0%)	
Diabetes					0.07
Yes (SR/RR)	67 (5.1%)	49 (6.4%)	86 (5.2%)^	31 (5.6%)	
Yes (fasting glucose only)	43 (3.3%)	31 (4.0%)	74 (4.5%)	36 (6.6%)	
No	1209 (92%)	690 (90%)	1490 (90%)	480 (88%)	
Systolic BP (sitting, mm/Hg)*	153.2 (23.9)	155.4 (23.9)	154.6 (24.0)	153.3 (25.4)	0.20
Missing	114 (9%)	150 (20%)	141 (9%)	16 (3%)	
LDL cholesterol (mmol/l)	4.2 (1.1)	4.2 (1.1)	4.1 (1.1)	4.1 (1.1)	0.21
Missing	155 (12%)	186 (24%)	161 (10%)	22 (4%)	
Waist circumference (mm)*	861.0 (119.3)	868.0 (120.7)	861.9 (122.9)	860.1 (125.8)	0.63
Missing	84 (6%)	126 (18%)	123 (8%)	3 (1%)	
Obesity					0.29
Yes	430 (33%)	341 (44%)	565 (34%)	163 (30%)	
No	813 (62%)	296 (39%)	967 (59%)	382 (70%)	
Missing	76 (6%)	133 (17%)	118 (7%)	2 (< 1%)	
Leg length (mm)	763.5 (41.4)	753.4 (42.9)	756.1 (40.2)	752.2 (39.5)	< 0.0005
Missing	75 (6%)	133 (2%)	118 (7%)	2 (< 1%)	
Family history of CVD**	750 (57%)	431 (56%)	893 (54%)	300 (55%)	0.49
Alcohol intake					< 0.0005
Most days	253 (19%)	127 (17%)	276 (17%)	58 (11%)	
Socially^$^	819 (62%)	439 (57%)	970 (59%)	319 (58%)	
Never	166 (13%)	133 (17%)	239 (15%)	109 (20%)	
Missing	81 (6%)	71 (9%)	165 (10%)	61 (11%)	
Physically inactive^$^	739 (56%)	510 (66%)	935 (57%)	285 (52%)	< 0.0005
Missing	149 (11%)	90 (9%)	214 (13%)	97 (18%)	
Limited fruit/veg intake^$$^	33 (2%)	44 (4%)	93 (3%)	40 (4%)	< 0.0005
Missing	129 (10%)	83 (11%)	242 (15%)	90 (17%)	
Carstairs index based on GB population data (mean (SD))	-0.9 (2.4)	0.6 (2.7)	0.5 (3.3)	2.67 (3.4)	< 0.0005
Missing	24 (2%)	1 (< 1%)	8 (1%)	36 (7%)	
Life-course SEP (median (25^th^, 75^th ^centiles))	4 (2, 5)	4 (3, 6)	4 (2, 6)	5 (3, 6)	< 0.0005
Missing^£^	305 (23%)	181 (24%)	452 (27%)	162 (30%)	
CVD medication	487 (37%)	252 (33%)	673 (41%)	262 (48%)	< 0.0005
Non-missing for all covariates	700 (53%)	382 (50%)	881 (53%)	293 (54%)	0.3

Note: Figures given in parentheses are percentages of the baseline responders unless otherwise stated

^§ ^Alive at time of mailing

* Mean of measurements

** Sibling or parent had stroke or heart attack (self-report)

^$ ^Those answering weekends only/once or twice a month/special occasions

^$ ^Response to nurse-led examination question (not questionnaire)

^$$ ^Missing where either summer fruit intake or winter fruit intake response is missing.

^£ ^Missing at least one of the ten components of SEP. Missingness of individual components ranged from 2.5-14.4%.

^ Includes one individual with diabetes indicated by diabetic medicines only

^^ From chi-squared test for binary variables, linear regression for continuous variables, and ordered logistic regression for ordinal variables (life-course SEP). Analyses carried out on non-missing values only. Analysis of diabetes variable combined all "yes" categories.

The impact of missing values in covariates was investigated using multiple imputed datasets, generated using chained equations (with 10 cycles of regression switching). Ten datasets were imputed using all other factors (including age and region) to impute missing values. Log survival time and binary indicator for event were also included as predictors [31]. Linear regression was used to impute missing values for continuous variables (SBP, LDLc, waist circumference, leg length, Carstairs index, age at baseline), multinomial logistic regression for categorical variables (alcohol intake, smoking status, region), and logistic regression for binary variables (diabetes, obesity, family history, limited physical activity, limited fruit intake, CVD medication, life-course socioeconomic position components). For the life-course socioeconomic position measure, imputations were made for the ten components separately. Clustering by town is accounted for in all analyses through the use of robust standard errors.

## Results

The characteristics of 4286 baseline responders are given in Table 1 by region. Missing data for the majority of factors was <10%. However, missingness for data collected at clinical examination varied by region, with the Midlands/Wales having a greater proportion of missing values for these factors. Socioeconomic position exhibited more missing values than other variables since it was derived from ten other variables.

In total there were 549 deaths in baseline responders (12.8%) in the follow-up period. Of these, 150 were in South England (11.4% of baseline responders), 117 in Midlands/Wales (15.2%), 210 in North England (12.7%) and 72 in Scotland (13.2%). CVD prevalence at baseline and incidence over the seven year follow-up period for the 4826 responders at baseline is given in Table 2 by region. As reported previously, baseline prevalence of CVD was highest in Scotland, and lowest in South England.

**Table 2:** CVD prevalence and incidence risk percent by region

	South England	Midlands/ Wales	North England	Scotland
Number of baseline responders	1319	770	1650	547
Prevalent CVD at baseline (% of baseline responders)	213 (16.1%)	177 (23.0%)	320 (19.4%)	135 (24.7%)

**Incident CVD - non-fatal**				
If prevalent disease at baseline (% those with prevalent disease)*	5 (2.4%)	7 (4.0%)	16 (5.0%)	4 (3.0%)
If no prevalent disease at baseline (% those without prevalent disease)	102 (9.2%)	47 (7.9%)	114 (8.6%)	38 (9.2%)
*Total non-fatal events*	*107 (8.1%)*	*54 (7.0%)*	*130 (7.9%)*	*42 (7.7%)*
**Incident CVD - fatal**				
If prevalent disease at baseline (% those with prevalent disease)	14 (6.6%)	16 (9.0%)	40 (12.5%)	10 (7.4%)
If no prevalent disease at baseline (% those without prevalent disease)	32 (2.9%)	21 (3.5%)	40 (3.0%)	17 (4.1%)
*Total fatal events*	*46 (3.5%)*	*37 (4.8%)*	*80 (4.8%)*	*27 (4.9%)*

Incident CVD - fatal/non-fatal combined**				
If prevalent disease at baseline (% those with prevalent disease)	19 (8.9%)	21 (11.9%)	55 (17.1%)	14 (10.4%)
If no prevalent disease at baseline (% those without prevalent disease)	129 (11.7%)	62 (10.5%)	148 (11.1%)	54 (13.1%)
*Total incident events*	*148 (11.2%)*	*83 (10.8%)*	*203 (12.3%)*	*68 (12.4%)*

* Only applies to MI and stroke events (angina and unstable angina cannot be both prevalent and incident).

** Fatal and non-fatal events may sum to greater than the combined events since some individuals have both fatal and non-fatal CVD events (but the combined numbers only include the first of these, i.e. only include the non-fatal event)

Results of analysis for all incident events (with or without prevalent disease) are given in Table 3. The estimates based on the observed data for all events combined suggest that compared to South England, incidence is lower in all other regions, although these estimates are imprecise; HR (after adjustment for risk factors) for Midlands/Wales 0.66 (95% CI 0.43 to 1.00), for North England 0.87 (95% CI 0.64 to 1.19) and for Scotland 0.75 (95% CI 0.47 to 1.19)). As has already been noted, Midlands/Wales exhibited a larger proportion of missing responses at baseline for the covariates of interest. Analysis of the imputed datasets suggests that accounting for the missing data explained much of the difference seen between each of the regions and South England, although the point estimate for the Midlands/Wales still suggests lower CVD incidence compared to South England (HR 0.85 (95% CI 0.64 to 1.12)).

**Table 3:** Hazard ratios for region comparisons in Cox regression models of CVD incidence and fatalities

	N	South England	Midlands/ Wales	North England	Scotland
**Hazard ratios for incident non-fatal CVD events (95% CI)**					
					
***Using subset with no missing values for any considered factor***					
Model 1	2253*	1	0.79 (0.50, 1.26)	0.97 (0.69, 1.37)	0.70 (0.41, 1.19)
Model 2**	2253*	1	0.71 (0.44, 1.14)	0.87 (0.61, 1.25)	0.62 (0.35, 1.12)
					
***Using imputed data for missing values (10 imputations)***					
Model 1	4281*^$^	1	0.64 (0.63, 1.23)	1.06 (0.82, 1.36)	0.92 (0.64, 1.33)
Model 2	4281*^$^	1	0.82 (0.58, 1.15)	0.99 (0.76, 1.29)	0.86 (0.58, 1.28)

**Hazard ratios for incident fatal CVD events (95% CI)**					
					
***Using subset with no missing values for any considered factor***					
Model 1	2256	1	0.83 (0.38, 1.83)	1.19 (0.67, 2.10)	1.59 (0.78, 3.25)
Model 2	2256	1	0.63 (0.28, 1.41)	0.98 (0.54, 1.76)	1.07 (0.48, 2.37)
					
***Using imputed data for missing values (10 imputations)***					
Model 1	4284^$^	1	1.41 (0.91, 2.18)	1.53 (1.06, 2.20)	1.63 (1.01, 2.63)
Model 2	4284^$^	1	1.13 (0.72, 1.77)	1.19 (0.82, 1.74)	1.09 (0.66, 1.82)


**Hazard ratios for combined incident fatal/non-fatal CVD events (95% CI)**					
					
***Using subset with no missing values for any considered factor***					
Model 1	2253*	1	0.77 (0.51, 1.16)	1.01 (0.75, 1.36)	0.92 (0.60, 1.41)
Model 2**	2253*	1	0.66 (0.43, 1.00)	0.87 (0.64, 1.19)	0.75 (0.47, 1.19)
					
***Using imputed data for missing values (10 imputations)***					
Model 1	4281*^$^	1	0.98 (0.75, 1.28)	1.20 (0.97, 1.48)	1.14 (0.85, 1.53)
Model 2	4281*^$^	1	0.85 (0.64, 1.12)	1.05 (0.84, 1.31)	0.93 (0.68, 1.27)

* excludes 3 with event on date of baseline questionnaire

^$ ^excludes 1 with missing date of birth and 1 with missing date for baseline questionnaire

** some evidence for violation of proportional hazards (global test in non-fatal Model 2a, p = 0.04; in combined Model 2a, p = 0.03)

*Model 1*: unadjusted

*Model 2*: adjusted for risk factors as described in Table 1 (except diabetes, where all cases were merged to give a binary variable)

Analyses carried out separately for fatal and non-fatal incident CVD provides further insight into the nature of regional variations. The incidence of fatal CVD events only was, after adjusting for all considered covariates and missing data, higher in all the regions compared to South England: Midlands/Wales HR 1.13 (95% 0.72 to 1.77), North England HR 1.19 (95% CI 0.82 to 1.74), Scotland HR 1.09 (95% CI 0.66 to 1.82). In contrast, incidence of non-fatal events was lower in the Midland/Wales (HR 0.82 (95% CI 0.58 to 1.15) and Scotland (HR 0.86 (95% CI 0.58 to 1.28) compared to South England (adjusting for covariates and missing data).

**Figure 2:**
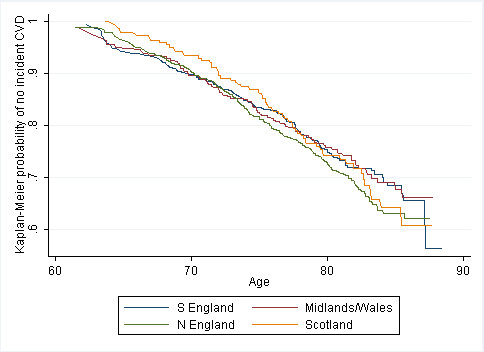
**Unadjusted Kaplan-Meier survival function for incident CVD events (whole cohort, irrespective of baseline CVD prevalence)**. All observed data (n = 4281).

**Figure 3:**
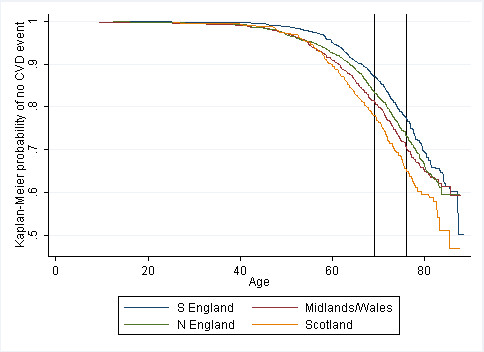
**Unadjusted Kaplan-Meier survival function for all CVD events (prevalent and incident combined)**. First line shows mean age at baseline; second line shows mean age at seven-year follow-up. All observed data (n = 4285).

The Kaplan-Meier survival functions by region for CVD incidence are shown in Figure 2. A similar figure showing prevalent and incident events combined is presented in Figure 3. This illustrates that although there are regional differences in CVD early in adult life [11], as supported by analysis of CVD prevalence at baseline in the BWHHS [11], there is little further divergence in later life. This helps explain why an analysis of CVD incidence occurring after age 69 (the mean age at baseline in the BWHHS) does not demonstrate strong regional differences.

## Discussion

Evidence from the BWHHS published previously indicated that region was associated with CVD prevalence over and above variation explained by known risk factors, with South England having the lowest prevalence [11]. Results here based on seven-year follow-up data suggest that regional variation in CVD incidence beyond baseline (i.e. beyond the mean age of 69) exhibits a very different pattern to that seen for CVD prevalence in this population. For CVD incidence, much of the difference between regions was explained by imputing missing covariate values, with hazard ratios for the Midlands/Wales, North England and Scotland generally attenuated towards no difference in incidence compared to south England. However, Kaplan-Meier survival functions showing prevalence and incidence combined suggest that regional differences begin at around age 55, and are prominent by age 69, when the baseline study was conducted. However, in the subsequent period between baseline and seven-year follow-up, there is no *further *divergence by region, although the differences by region remain at age 76 (corresponding to the mean age at seven-year follow-up). These results suggest that the regional differences in CVD occur at earlier ages; while examination of CVD prevalence is likely to show regional variation across a range of ages, regional differences in CVD incidence may only be apparent at younger ages. This possibility is supported by the original analysis of the regional differences in incidence data among men at much younger ages (40-59), which showed marked north-south variation [18].

### Explanation of findings

The absence of continued *divergence *of the regions in terms of CVD may arise for a number of reasons. There may be regional differences in terms of early diagnosis or treatment of conditions such as angina, which may arise due to differences in health care provision or access to/use of health care services. Such differences in diagnosis would influence the cohort at earlier ages, but may have relatively little impact later on. If this were the case, regions exhibiting higher CVD at younger ages may actually represent better clinical practice (resulting in earlier diagnosis and improved survival following a CVD event). However, since these results are adjusted for CVD medication, it seems unlikely that differences in early detection account for regional differences in CVD incidence. The potential impact of improved healthcare on survival following a CVD event implies that regions with higher non-fatal CVD incidence also show lower fatal CVD incidence (as observed here in South England).

Survivorship may also play a role. Although prevalent non-fatal CVD events are included in analysis, fatal CVD events (and other cause mortality) occurring prior to baseline are not, since by definition an individual must be alive to participate in the study. Those still alive at the start of the study will thus be a healthier group. The impact of this survivorship may vary between regions since mortality (CVD and other causes) may differ between regions prior to baseline. This would imply that regions exhibiting lower CVD prevalence in fact represent a group from which more of the least healthy individuals have already died.

It may also be possible that a different type of individual is at risk of early CVD (for example, before age 60). If this early onset CVD differed by region, but later onset CVD was more similar between regions, this would be consistent with the observations described here. Lastly, it is possible that there are regional differences in response to recent guidance on the management of cardiovascular risk factors and both primary and secondary prevention.

### Consistency with other studies of geographic variation

Regional differences in cardiovascular disease incidence and mortality have been reported for a number of other countries, including the United Kingdom [32], the United States [19,33], Canada [14], Finland [20], Sweden [10], France [13,16], and across Europe [15]. Some of these studies have sought to simply identify and describe regional differences. Others have attempted to use known risk factors to explain the regional variations in CVD [19,33], although in some cases analysis was performed on aggregated data (i.e. individual patient data were not available) [14]. A study of regional differences in 12-year stroke incidence in the United States [33] showed that the differences could largely, but not entirely, be explained by known risk factors in a cohort of 7000 men and women aged 45-74. More prominent regional differences remained after adjustment in the women. This supports the results from the BWHHS cohort, which suggest that known risk factors do not account for all observed geographical variation in CVD. A further study in the United States has reported regional differences in stroke and fatal CVD, but not MI or non-fatal/fatal CVD combined [19]. Adjustment for known risk factors in this study further exaggerated regional differences. However, restriction of participants to physicians and exclusion of 16% of subjects with missing baseline data raises questions regarding the generalisability of these results. Finally, the BRHS has provided much of the UK evidence for regional variations in CVD in men. This work has provided evidence that for men aged 40-59 at baseline, around 75% of the between-town variation in 5-, 10-, and 15-year incidence of coronary heart disease can be explained by smoking, systolic blood pressure, exercise, social class, and height [18]. This again suggests that most - but not all - observed regional variations can be explained by classical risk factors.

### Limitations and strengths of the study

One of the potential weaknesses of this analysis is missing data. This problem has two components, since there are missing data in the CVD risk factors included as covariates in the Cox models, and also non-response at each of the three follow-up time-points. The former is addressed through the use of multiple imputation, which assumes that the missing covariate values are missing at random (MAR) - that the missing values depend on observed values of other variables, but not on the missing values themselves. Given the well-documented strong relationships between many of the covariates of interest here, this would seem a generally reasonable assumption. Furthermore, imputation based on non-missing covariates is reasonable since individuals do not generally have many missing covariates (of those with at least one missing value at baseline, 1839/1976 (93%) have at least 9/15 non-missing values). The results from analysis of the multiply imputed datasets suggest that accounting for the missing data explains some of the observed differences between regions.

The second component of the missing data issue is non-response to either the baseline or follow-up questionnaires. Non-response to the follow-up questionnaires is not addressed here, since it is anticipated that the majority of self-report CVD events missed as a result of this non-response would be picked up by record review (only 5% of individuals are lost to follow-up from both sources). Furthermore, there is little evidence of a difference in response by region at either three-year (χ^2 ^p = 0.09) or seven-year follow-up (χ^2 ^p = 0.18). Non-response at baseline however is more pronounced than at subsequent waves (59% response across all regions) and there is strong evidence for a difference in response at baseline by region (χ^2 ^p < 0.0005). Since non-responders at baseline are excluded from all analyses, this has implications for bias and generalisability of the results presented here. If regions with higher non-response at baseline actually have higher CVD incidence, the large number of non-responders (those at likely to be at highest risk of CVD) would render estimates of regional differences conservative.

## Conclusions

To conclude, the British Women's Heart and Health Study provides a strong platform for regional analysis, with randomly selected subjects from 23 towns representing areas throughout the UK. Furthermore, the study includes over 4000 women with a mean of seven years follow-up. These results show that in women, regional differences in CVD (over and above those explained by known risk factors) occur relatively early in life, but do not continue to diverge in later life. Analysis of the incidence of fatal and non-fatal CVD combined showed different patterns of variation between the regions, suggesting regional differences in prognosis following a CVD event. These findings imply that in women, efforts to characterise and standardise regional differences in CVD-related healthcare should be targeted at those under 70. Further follow-up of the BWHHS is required in order to increase numbers of events and thus increase the precision of the estimates obtained in these analyses. Further work is also needed to investigate potential explanations for the apparent improvement in CVD outcomes observed in South England compared to other regions.

## Competing interests

The authors declare that they have no competing interests.

## Authors' contributions

LK carried out the analysis and wrote and revised the manuscript. SE, DL and CC helped draft and revise the manuscript critically for important intellectual content. All authors read and approved the final manuscript.

## Pre-publication history

The pre-publication history for this paper can be accessed here:

http://www.biomedcentral.com/1471-2458/10/696/prepub

## Supplementary Material

Additional file 1 **Baseline questionnaire**. A self administered questionnaire about lifestyle and medical history used at the baseline visit. Click here for file

Additional file 2 **3-year follow-up questionnaire (long form)**. A self administered questionnaire about lifestyle and medical history which was sent out in March 2003. Click here for file

Additional file 3 **3-year follow-up questionnaire (short form)**. A self administered questionnaire about lifestyle and medical history which was sent out in March 2003. Click here for file

Additional file 4 **7-year follow-up questionnaire**. A third self-administered postal questionnaire was sent in 2007. This was about health and lifestyle to see what was changed, and what has stayed the same since the last survey in 2003. Click here for file
