# Supplementary material for: Geographical variation in cardiovascular incidence: results from the British Women's Heart and Health Study
Source: BMC Public Health. 2010 Nov 15;10:696. doi: 10.1186/1471-2458-10-696 (PMC2996371; doi:10.1186/1471-2458-10-696)
Supplement: Additional file 1 — Baseline questionnaire. A self administered questionnaire about lifestyle and medical history used at the baseline visit. [file 1471-2458-10-696-S1.PDF]

Questionnaire Number

Study Number :

Town:

BRITISH WOMEN'S HEART & HEALTH STUDY

BASELINE SURVEY

- This questionnaire asks about your health, your life-style and your social background. This will give vital information for our research.
- Most questions can be answered simply by ticking the correct box ☑
- All the information collected will be treated as strictly confidential.
- **Please complete the form today, or as soon as possible, and return in the reply paid envelope.** If you have any difficulties with the questions, please phone us on 0117 928 7327 and leave your phone number so that we can call you back and answer your queries.

Thank you for your help.

British Womens' Heart & Health Study
Department of Social Medicine
Canynge Hall
Whiteladies Road
Bristol BS8 2PR

Please give the following information to help us contact you in the future.

1.0 Your telephone number ______________________________

1.2 Your date of birth | | | | | | |
Day Month Year

1.3 Today's date | | | | | | |
Day Month Year

1.4 Your maiden name, if you are married, divorced or widowed:

____

Name and address of family member or friend we could contact only if necessary:

1.5 Surname ______________________________

1.6 First name ______________________________

1.7 Address ______________________________

____

____

____

1.8 Post code: ______________________________

1.9 Telephone Number: ______________________________

Please answer the following questions by filling in the appropriate box with a tick ☑ or writing the answer in the space provided.

2.0 **Health at present**

How would you describe your health at present ?

Excellent	1	☐
Good	☐	2
Fair	☐	3
Poor	☐	4

3.0 **Conditions affecting the heart or circulation**

Have you ever been told by a doctor that you have or have had any of the following conditions ?

	Yes	No		If Yes, please give year when first diagnosed, if possible
3.1 Heart attack (coronary thrombosis or myocardial infarction)	☐	☐ [illegible]		19________________
3.2 Heart failure	☐	☐	3.8	19________________
3.3 Angina	☐	☐	3.9	19________________
3.4 Other heart trouble	☐	☐ [illegible]		19________________
3.5 High blood pressure	☐	☐	3.11	19________________
3.6 Stroke	☐	☐	3.12	19________________

4.0 **Cancers**

4.1 Have you ever been told by a doctor that you have or have had a cancer?

Yes ☐ No ☐

If yes, please state what kind of cancer(s):

	office use	Please give year when first diagnosed
4.2___________________________	☐☐☐	4.5 19_____________________
4.3___________________________	☐☐☐	4.6 19 _____________________

4.4___________________________ ☐☐☐ 4.7 19 ______________________

Please answer the following questions by filling in the appropriate box with a tick ☑ or writing the answer in the space provided.

5.0 **Other medical conditions**

Have you ever been told by a doctor that you have or have had any of the following conditions?

	Yes	No	Please give year when first diagnosed, if possible
5.1 Asthma	☐	☐	5.11 19_______________________
5.2 Bronchitis	☐	☐	5.12 19_______________________
5.3 Depression	☐	☐	5.13 19_______________________
5.4 Gastric, peptic or duodenal ulcer	☐	☐	5.14 19_______________________
5.5 Gout	☐	☐	5.15 19_______________________
5.6 Gall bladder disease	☐	☐	5.16 19_______________________
5.7 Osteoporosis	☐	☐	5.17 19_______________________
5.8 Thyroid disease	☐	☐	5.18 19_______________________
5.9 Cataract	☐	☐	5.19 19_______________________
5.10 Glaucoma	☐	☐	5.20 19 _______________________

6.0 **Falls and Fractures**

		Yes	No
6.1	Have you had a fall in the last 12 months ?	☐	☐

6.2 *If Yes,* how many times ? ___________

		Yes	No
6.3	Did you have medical attention for any of these falls ?	☐	☐

Fractures: Yes No Please give year

6.4 Have your ever fractured or broken your hip? ☐ (☐ 19________

6.5 or, your wrist? ☐ ☐ 6.7 19________

Please answer the following questions by filling in the appropriate box with a tick ☑ or writing the answer in the space provided.

7.0 **Arthritis**

	Yes	No
7.1 Have you ever been told by a doctor that you have or have had arthritis?	☐	☐

If Yes, please state what kind of arthritis:

	Yes	No	Don't know		Please give year first diagnosed
7.2 rheumatoid arthritis	☐	☐	☐	7.5	19______________
7.3 osteoarthritis	☐	☐	☐	7.6	19______________
7.4 other type of arthritis	☐	☐	7. ☐	19______________	

Which joints are or were affected?

		Yes	No
7.8	hips	☐	☐
7.9	knees/ankles	☐	☐
7.10	shoulders	☐	☐
7.11	hands/fingers	☐	☐
7.12	back/spine	☐	☐

8.0 **Operations**

	Yes	No
8.1 Have you ever had an operation(s)?	☐	☐

If Yes, please give details including the year: office use Please give year of operation(s)

8.2 ______________________________ ☐☐☐ 8.5 19______________

8.3 ______________________________ ☐☐☐ 8.6 19______________

☐☐☐

8.4 ________________________________ 8.7 19_______________

Please list any other operations here:

Please answer the following questions by filling in the appropriate box with a tick ☑ or writing the answer in the space provided.

9.0 **Hearing and vision**

Do you have trouble with

	Yes	No
9.1 your hearing	☐	☐
9.2 your eyesight *(not simply needing specs)*	☐	☐

If Yes, please give details: office use

9.3 Hearing____ ☐☐

9.4 Vision____ ☐☐

10.0 **Diabetes**

	Yes	No	Don't know
10.1 Has anyone in your close family (your parents, brothers, sisters) ever had diabetes ?	☐	☐	☐

	Yes	No	Don't know	Year first diagnosed
10.2 Have you ever been told by a doctor that you have or have had diabetes?	☐	☐	☐	10.3 19____________
If Yes:				
10.4 Are you on a regular diet for your diabetes ?	☐	☐	☐	
10.5 Are you on regular tablets for your diabetes?	☐	☐	☐	
10.6 Are you on regular treatment with insulin?	☐	☐	☐	
10.7 Do you attend a hospital or GP diabetic clinic?	☐	☐	☐	

11.0 **Breathlessness**

		Yes	No	Unable
11.1	Do you get short of breath walking with other people of your own age on level ground?	☐	☐	☐
11.2	On walking uphill or stairs do you get more breathless than people of your own age?	☐	☐	☐
11.3	Do you ever have to stop walking because of breathlessness ?	☐	☐	☐

Please answer the following questions by filling in the appropriate box with a tick ☑ or writing the answer in the space provided.

12.0 **Leg pain**

		Yes	No	Unable
12.1	Do you ever get pain or discomfort in your leg, thighs or buttocks when you walk?	☐	☐	☐

If, No or Unable to walk go on to question 13 "Ankle swelling" on next page.

		Yes	No
12.2	Do you know the cause of the pain?	☐	☐

12.3 *If Yes*, what is the cause? ______________________________ office use ☐☐

		Yes	No
12.4	Does this pain ever begin when you are standing still or sitting?	☐	☐

		Yes	No	Unable
12.5	Do you get the pain if you walk up hill or hurry?	☐	☐	☐
12.6	Do you get the pain walking at an ordinary pace on the level?	☐	☐	☐

12.7 What happens to the pain if you stand still?

Usually continues more than 10 minutes 1 ☐ Usually disappears in <10 minutes 2 ☐

12.8 Where do you get the pain? **Shade regions affected**

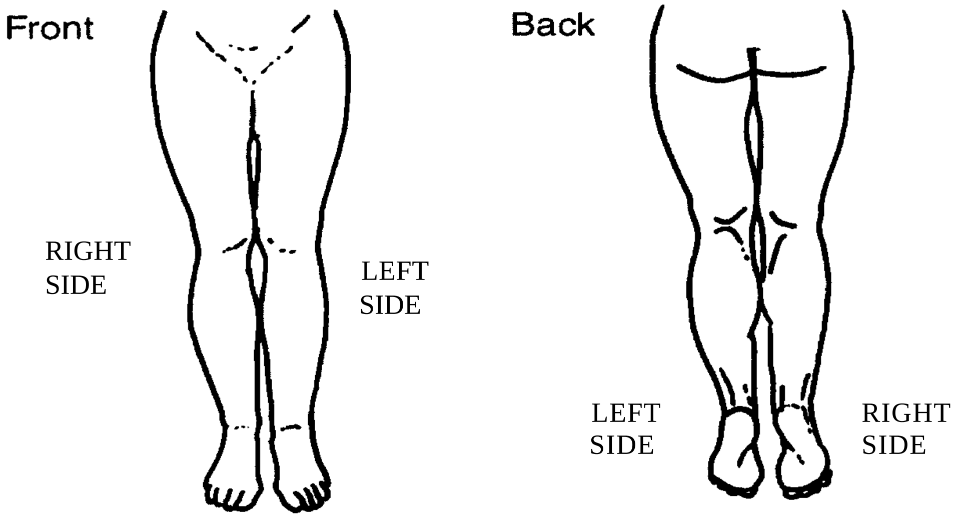

office use

☐☐

Please answer the following questions by filling in the appropriate box with a tick ☑ or writing the answer in the space provided.

13.0 **Ankle swelling**	Yes	No	Don't know
13.1 Do your ankles swell up regularly ?	☐	☐	☐
13.2 *If Yes*, is this because of varicose veins?	☐	☐	☐

14.0 **Cough and Wheeze**			
	Yes	No	Don't know
14.1 Do you usually bring up phlegm (spit) from your chest first thing in the morning in the winter ?	☐	☐	☐
14.2 *If Yes*, do you bring up phlegm like this on most days for as much as 3 months in the winter each year?	☐	☐	☐
	Yes, once	Yes, more often	Never
14.3 In the past 4 years have you ever had a period of increased cough and phlegm lasting for 3 weeks or more?	☐	☐	☐
	Yes	No	Don't know
14.4 Does your chest ever sound wheezy or whistling?	☐	☐	☐
	Yes	No	Don't know
14.5 *If Yes,* does this happen on most days or nights?	☐	☐	☐

15.0 **Treatment with aspirin**	Yes	No
15.1 Do you take aspirin regularly?	☐	☐

If Yes,

15.2 Is this on doctor's advice? ☐ ☐

15.3 When did you start taking aspirin regularly ? 19 ____________

15.4 On how many days each week do you take aspirin?

daily ☐ 1 alternate days ☐ 2 other ☐ 3

15.5 What dose of aspirin do you take each day that you take it?

75mg/1/2junior ☐ 1 125mg/junior ☐ 2 300mg/adult ☐ 3 other ☐ 4

15.6 For what condition are you taking aspirin ?

office use

Please state ______________________________________ ☐☐

Please answer the following questions by filling in the appropriate box with a tick ☑ or writing the answer in the space provided.

16.0 **Hormone replacement therapy (HRT)**

	Yes	No	Don't know
16.1 Have you ever taken HRT?	☐	☐	☐

If Yes,

16.2 Are you still taking it? ☐ ☐

16.3 How long have you (or did you) taken it ? __________________years

If stopped now,

16.4 How long ago did you stop taking it? ________________________ years

office use

16.5 Which preparation do/did you use? ___________________________ ☐☐

17.0 **Vitamin or mineral tablets**

	Yes	No
17.1 Do you take any vitamin or mineral tablets or supplements?	☐	☐

office use

If Yes, please give details: __________________________________17.2 ☐☐

18.0 **Weight**

18.1 What is your present weight ? __________Stones ________ Pounds

18.2 What is your current dress size? __________

18.3 What was your weight as a young woman aged 21? __________Stones ________ Pounds

18.4 What was your dress size as a young woman aged 21?__________

18.5 Have you dieted during your adult life?

1 ☐ yes, regularly   2 ☐ yes, on and off   3 ☐ no

18.6 Has your weight changed in the last four years?

☐ 1 not changed   ☐ 2 increased   ☐ 3 decreased   ☐ 4 up/down   ☐ 5 don't know

Please answer the following questions by filling in the appropriate box with a tick ☑ or writing the answer in the space provided.

Weight (continued)

18.7 *If your weight has increased or decreased in the last 4 years*,

how much weight have you gained or lost? _______stones _______ lbs

	Yes	No
18.8 *If you have lost weight*, was this intentional? (eg. dieting)	☐	☐

19.0 **Smoking**

	Yes	No	
19.1 Have you ever smoked cigarettes regularly (at least 1/day)?	☐	☐	**If No, go to 19.8**
If Yes:			
19.2 Do you smoke cigarettes at present?	☐	☐	

If Yes:

19.3 How many cigarettes do you smoke a day? __________ cigarettes

If No, go to 19.6

19.4 If hand-rolled, how much tobacco do you use a week ?

______________ounces   19.5 _____________grams

19.6 How old were you when you started smoking regularly? _______years ←

19.7 Have you changed your cigarette smoking habits over the last 4 years ?

☐ 1 Yes, increased   ☐ 2 Yes, cut down   ☐ 3 Yes, given up   ☐ 4 No

19.8 Do you currently smoke tobacco in any other form (e.g. pipe, cigar)?   Yes ☐   No ☐ ←

If No,   Yes   No

19.9 Have you ever regularly done so?   ☐   ☐

Please answer the following questions by filling in the appropriate box with a tick ☑ or writing the answer in the space provided.

Smoking (continued)

For ex-smokers

19.10 Were you previously a regular cigarette smoker?   Yes ☐   No ☐

If Yes,

19.11 How many cigarettes did you usually smoke each day ?_____________cigarettes

19.12 At what age did you give up? ______________ years old

19.13 Why did you give up? Tick one main reason only.

☐ 1 Personal choice   ☐ 2 Financial reasons   ☐ 3 Health precaution

☐ 4 Doctor's advice   ☐ 5 Illness or ill-health   ☐ 6 Other reasons

19.14 Does/did your husband/partner smoke cigarettes?

☐ 1   ☐ 2   ☐ 3   ☐ 4

Yes	No	Ex-smoker	Not applicable

20.0 **Alcohol Intake**

20.1 Would you describe your present alcohol intake as

- Daily/most days ☐ 1
- Weekends only ☐ 2
- Once or twice a month 3 ☐
- Special occasions ☐ 4
- Never ☐ 5

20.2 One drink is **HALF** a pint of beer, a **SINGLE** whisky, gin etc., or **ONE GLASS** of wine or sherry. How much do you usually drink each day ?

- More than 6 drinks a day ☐ 1
- 3-6 drinks a day ☐ 2
- 2 drinks a day or less ☐ 3
- None ☐ 4

20.3 How many alcoholic drinks do you take during an average **week**? _________drinks

Please answer the following questions by filling in the appropriate box with a tick ☑ or writing the answer in the space provided.

Alcohol (continued)

20.4 What type of drink do you usually take ?

- Beers, Lagers ☐ 1
- Sherry, wine ☐ 2
- Spirits ☐ 3
- Variety of beer, wines or spirits ☐ 4
- Low alcohol drinks ☐ 5

		Yes	No	If **Yes**, glasses per week
20.5	Do you drink white wine?	☐	☐	_____________glasses/week
20.6	Do you drink red wine?	☐	☐	_____________glasses/week

20.7 Have you changed your alcohol intake in the last four years?

- No ☐ 1
- Yes, increased ☐ 2

Yes, cut down ☐ 3

Yes, given up ☐ 4

*If you have **CUT DOWN** or **GIVEN UP***

20.8 Was this due to: Tick one main reason only.

☐1 Personal choice ☐2 Financial reasons ☐3 Health precaution

☐4 Doctor's advice ☐5 Illness or ill-health ☐6 On medication ☐7 Other reasons

For those not drinking at present

		Yes	No
20.9	Did you drink in the past ?	☐	☐

If Yes,

20.10 would you describe your previous alcohol intake as

Daily/most days ☐ 1

Weekends only ☐ 2

Once or twice a month or special occasions ☐ 3

20.11 How many alcoholic drinks did you take during an average week? ________ drinks/week

20.12 How many years ago did you stop? ____________________years ago

Please answer the following questions by filling in the appropriate box with a tick ☑ or writing the answer in the space provided.

21.0 **Your diet**

		Yes	No
21.1	Do you eat any special diet?	☐	☐

21.2 *If Yes*, please specify

☐ 1 low fat high fibre ☐ 2 vegetarian ☐3 diabetic ☐ 4 sliming/low calorie ☐5 other ☐6

21.3 What kind of bread do you eat?

☐ 1 White ☐ 2 Brown ☐ 3 Wholemeal ☐ 4 Various

21.4 Spreading fat: What kind do you use at home?

☐ 1 Butter ☐2 Margarine ☐ 3 Margarine ☐ 4 Low calorie ☐ 5 Various ☐ 6 None

	(Hard)	(Soft)	spread<br>(e.g. Delight)

How often do you eat the following foods? (Please tick the appropriate box for each food item)

	1	2	3	4	5	6
	More than once a day	Once a day	Most days	One or two days a week	Less than once a week	Never
21.5 Fresh fruit summer						
21.6 Fresh fruit winter						
21.7 Salads in summer						
21.8 Salads in winter						
21.9 Green vegetables						
21.10 Fish (all kinds)						
21.11 Poultry (eg. chicken turkey)						
21.12 Red meat (eg. beef, pork, ham, bacon)						
21.13 Processed meat (eg. burgers, sausages, pies, pasties, pate)						
21.14 Cereals						
21.15 Nuts						
21.16 Cheese						

Please answer the following questions by filling in the appropriate box with a tick ☑ or writing the answer in the space provided.

Your diet (continued)

21.17 What kind of cooking fat do you usually use at home?

☐ 1 Lard, butter, animal fat
☐ 2 Vegetable oil
☐ 3 Olive oil
☐ 4 Various fats
☐ 5 Other fats

21.18 What type of milk do you usually use?

☐ 1 Full cream
☐ 2 Semi-skimmed
☐ 3 Skimmed
☐ 4 Dried
☐ 5 Tinned
☐ 6 None
☐ 7 Other

22.0 **Physical Activity**

22.1 Which of the following forms of transport do you use most often? Please tick only one box

☐1	☐2	☐3	☐4	☐ 5
Car	Public Transport	Cycle	Walk	Not applicable

22.2 Do you make regular journeys every day or most days either walking or cycling?

☐1	☐2	☐3	☐4
No	Walk	Cycle	Both

22.3 Which of the following best describes your usual walking pace?

☐1	☐2	☐3	☐4
Slow	Steady average	Fairly brisk	Fast (at least 4miles/hr)

22.4 *If you cycle regularly*, how long do you spend cycling in an average week? __________ hours/week

22.5 Do you take physical activity such as running, swimming, dancing, golf, tennis, squash, jogging, bowls?

☐1	☐2	☐3
No	Occasionally (less than monthly)	Frequently (once a month or more)

If you take part in these physical activities ***frequently,*** (once a month or more):
How many **times** a month on average do you take part in these activities?

22.6 Summer _______________ times/month

22.7 Winter ________________ times/month

Please answer the following questions by filling in the appropriate box with a tick ☑ or writing the answer in the space provided.

Physical activities (continued)

In a **typical week** during the past year, how many hours did you spend each week in the following activities? Write 0 if no activity.

Walking to work, shopping and leisure 22.8 Summer______ hours/week

22.9 Winter _______hours/week

Cycling, including to work and leisure 22.10 Summer______hours/week

	22.11	Winter	_______hours/week
Gardening, light eg. pruning, watering	22.12	Summer	______hours/week
	22.13	Winter	_______hours/week
Gardening, heavy eg. digging, mowing	22.14	Summer	______hours/week
	22.15	Winter	_______hours/week
Physical exercise eg. fitness, aerobics,	22.16	Summer	______hours/week
swimming, jogging, tennis	22.17	Winter	_______hours/week
DIY eg. on house, car	22.18		_______hours/week
Housework activities, light eg. cooking washing up, dusting	22.19		_______hours/week
Housework, heavy, eg. hoovering, floors window cleaning	22.20		_______hours/week

Please answer the following questions by filling in the appropriate box with a tick ☑ or writing the answer in the space provided.

Physical activity (continued)

22.21 In a **typical week** in the last year, did you do any of these activities vigorously enough to cause breathlessness, sweating or a faster heart beat? Yes ☐ No ☐

22.22 *If Yes*, for how many minutes each week did you perform vigorous activity?_______minutes/week

22.23 In a typical week in the last year, how many flights of stairs

do you climb a day? ______flights/day

22.24 Compared with your activity level of three years ago, are you doing

☐ 1 More ☐ 2 Same ☐ 3 Less

office use ☐

22.25 *If less*, please give the reason_____________________________________

22.26 Compared with other woman of your age, are you:

☐ 1 Much more active ☐ 2 More active ☐ 3 Similar ☐ 4 Less active ☐ 5 Much less active

23.0 **Your health overall**

Thinking about your health TODAY which of the following is the most applicable.

23.1 I have no pain or discomfort ☐ 1

I have moderate pain or discomfort ☐ 2

I have extreme pain or discomfort ☐ 3

23.2 I have no problems with performing my usual activities 1 ☐

I have some problems with performing my usual activities ☐ 2

I am unable to perform my usual activities ☐ 3

Please answer the following questions by filling in the appropriate box with a tick☑ or writing the answer in the space provided.

Your health overall (continued)

23.3 I have no problems with washing and dressing 1 ☐

I have some problems with washing and dressing ☐ 2

I am unable to wash and dress myself 3 ☐

☐

23.4 I have no problems in walking about ☐ 1

I have some problems in walking about 2 ☐

I am confined to a chair/wheelchair ☐ 3

23.5 I am not anxious or depressed ☐ 1

I am moderately anxious and/or depressed ☐ 2

I am extremely anxious and/or depressed ☐ 3

23.6 Compared to five years ago, is your memory

☐ 1	☐ 2	☐ 3	☐ 4	☐ 5
Improved	Same	Almost as good	Worse	Much worse

24.0 **<u>Disability</u>**

	Yes	No
24.1 Do you have any long-standing illness, disability or infirmity ?	☐	☐

(`long-standing' means anything which has troubled you over a period of time or is likely to do so)

<u>If Yes</u>

	Yes	No
24.2 Does this illness or disability limit your activities in any way?	☐	☐

24.3 What is the **<u>main</u>** medical problem causing this disability? If you have several medical problems, please give the most severe one.

____ office use ☐☐

	Yes	No
24.4 Do you receive a disability or other allowance for this?	☐	☐

Please answer the following questions by filling in the appropriate box with a tick ☑ or writing the answer in the space provided.

Disability (continued)

Do you currently have difficulty carrying out any of the following activities on your own as a result of a long term health or medical problems, or due to old age?

		Yes	No	Please give the year this first started	
24.5	Going up or down stairs	☐	☐	24.11	19_______
24.6	Bending down	☐	☐	2[illegible]12	19_______
24.7	Straightening up	☐	☐	24.13	19_______
24.8	Keeping your balance	☐	☐	24.14	19_______
24.9	Going out of the house	☐	☐	2[illegible]15	19_______
24.10	Walking 400 yards	☐	☐	24.16	19_______

Do you currently use any aids or appliances to help with day to day activities?

		Yes	No
24.17	Walking stick	☐	☐
24.18	Walking frame	☐	☐
24.19	Wheelchair	☐	☐
24.20	Toilet raised seat	☐	☐
24.21	Bath board/shower	☐	☐
24.22	Extra rails in bathroom	☐	☐
24.23	Stair lift	☐	☐

Please answer the following questions by filling in the appropriate box with a tick ☑ or writing the answer in the space provided.

Health problems

Is your present state of health causing problems with any of the following ?

		Yes	No
24.24	Job (paid employment)	☐	☐
24.25	Household chores	☐	☐
24.26	Social life	☐	☐
24.27	Sex life	☐	☐
24.28	Interests and hobbies	☐	☐
24.29	Holidays and outings	☐	☐
24.30	Family relationships	☐	☐

25.0 **<u>Your present circumstances</u>**

25.1 Are you:

☐ 1 Single ☐ 2 Married ☐ 3 Widowed ☐ 4 Divorced/separated ☐ 5 Other

25.2 Are you at present

living alone ☐ 1

living with a husband or partner ☐ 2

living with other family member(s) ☐ 3

living with other people 4 ☐

		Yes	No
25.3	Do you have a car available for use in your household ?	☐	☐

25.4 Your accommodation: are you an owner occupier ☐ 1

renting from a local authority ☐ 2

renting privately ☐ 3

other (please specify) ☐ 4

____

Please answer the following questions by filling in the appropriate box with a tick ☑ or writing the answer in the space provided.

Education and employment

25.5 How old were you when you finished full time education. ___________ years old

25.6 At present are you

- a housewife ☐ 1
- retired ☐ 2
- employed, full time ☐ 3
- employed, part time ☐ 4

25.7 If you are **retired**, is this due to

- normal retiring age 1 ☐
- early retirement, voluntary 2 ☐
- early retirement, compulsory ☐ 3
- illness/disability 4 ☐
- other reasons ☐ 5
- not applicable ☐ 6

25.8 If you are **retired**, please give the year in which you retired 19 ___________

25.9 What job have you done for the longest period of time ?

____ ☐ 25.10 ☐☐

25.11 Would you describe this work as

- Manual 1 ☐
- Non-Manual ☐ 2

Concerning your husband or partner:

25.12 Has your husband or partner ever suffered with any of the following? Please answer even if you are now widowed or divorced/separated.

	Yes	No
Heart attack	☐	☐
Stroke	☐	☐
Cancer	☐	☐

Please answer the following questions by filling in the appropriate box with a tick ☑ or writing the answer in the space provided.

Concerning your husband or partner (continued):

25.13 At present is your husband/partner
- retired ☐ 1
- employed, full time ☐ 2
- employed, part time ☐ 3
- unemployed, seeking work ☐ 4
- unemployed, not seeking work 5 ☐
- not applicable (eg. widowed) ☐ 6

25.14 If he is are **retired**, is this due to
- normal retiring age ☐ 1
- early retirement, voluntary ☐ 2
- early retirement, compulsory ☐ 3
- illness/disability 4 ☐
- other reasons ☐ 5
- not applicable ☐ 6

25.15 If he is retired, in which year did retired ? 19 ____________

25.16 If he is **unemployed**, is this due to
- redundancy ☐ 1
- illness/disability ☐ 2
- other reasons ☐ 3

25.17 What job has your husband or partner done for the longest period of time? Please answer even if he is now deceased, or you are now divorced or separated.

____ ☐ 25.18 ☐☐

25.19 Would you describe this work as
- Manual 1 ☐
- Non-Manual ☐ 2

Please answer the following questions by filling in the appropriate box with a tick ☑ or writing the answer in the space provided.

<u>Pensions</u>

25.20 What type of financial income do you (and your husband/partner) have or will you have on retirement ?

state pension <u>only</u>	1	☐
occupational pension, fixed amount	☐	2
occupational pension, index linked	☐	3
private pension	4	☐
occupational and private pensions	☐	5
don't know	☐	6

<u>Contact with relatives and friends</u>

How often do you see or speak to :-

Please tick the appropriate box in each row

		Every day 1	Every week 2	Every few months 3	Every year 4	Rarely or never 5	Does not apply 6
25.21	Your children						
25.22	Brothers/sisters						
25.23	Friends						
25.24	Neighbours						

Is the amount of contact you have with each of these:-

Please tick the appropriate box in each row

		Too little 1	About right 2	Too much 3	Does not apply 4
25.25	Your children				
25.26	Brothers/sisters				
25.27	Friends				

25.28	Neighbours				

Please answer the following questions by filling in the appropriate box with a tick ☑ or writing the answer in the space provided.

26.0 **Your earlier life and health**

Recent research suggests that your weight at birth may be important in later life. We need to ask you some questions about your early life.

26.1 How much did you weigh when you were born?

Write 00/00 if you don't know. __________lbs _________ozs

As a child, did the home you lived in longest have:

		Yes	No	Don't know
26.2	A bathroom	☐	☐	☐
26.3	Hot water	☐	☐	☐
26.4	Your own bedroom	☐	☐	☐
26.5	Use of a car	☐	☐	☐

Your periods

26.6 At what age did your periods **start** ? ______________________

26.7 At what age did your periods **stop** ? ______________________

26.8 Did your periods stop
naturally ☐ 1
because of an operation ☐ 2
(please give details) ________________________

office use
26.9 ☐

26.10 Have you ever taken the oral contraceptive pill ? Yes ☐ No ☐

26.11 *If Yes*, which type of pill did you take?
Combined pill 1 ☐
Progestogen only (mini-pill) 2 ☐
☐

Don't know 3

26.12 *If Yes*, for how long did you take it ? ________________years

26.13 In what year did you last take the pill ? 19____________

Please answer the following questions by filling in the appropriate box with a tick ☑ or writing the answer in the space provided.

27.0 **Your pregnancies**

27.1 How many pregnancies did you have? Give number _________

27.2 How many live births did you have? Give number _________

For you first born child, please give the following details: If no live births, please go to 27.7

27.3 Boy ☐ Girl ☐ 27.4 Born on time ☐ Early ☐ Late ☐

27.5 Birthweight _____ lbs _____ ozs

Did you have any of the following complications during any of your pregnancies?

		Yes	No
27.3	High Blood Pressure	☐	☐
27.4	Sugar in the urine	☐	☐
27.5	Diabetes	☐	☐
27.6	Swelling of the hands or feet	☐	☐
27.7	Pre-eclampsia	☐	☐

28.0 **Family history**

Your father

		Yes	No
28.1	Is your father still alive	☐	☐

If No, 28.2 How old was he when he died?___________years

28.3 What were you told was the cause of his death. Please tick only one cause.

Heart attack	☐	1	Other cancer	☐	6
High blood pressure	☐	2	Accident or injury	☐	7
Stroke	☐	3	Other cause	☐	8
	☐			☐	

Respiratory disease 4 Don't know 9

Cancer of lung ☐ 5

28.4 What job did your father do for the longest period of time? office use

____ ☐ 28.5 ☐☐

28.6 Would you describe this job as: Manual 1 Non manual ☐ 2 ☐

Please answer the following questions by filling in the appropriate box with a tick ☑ or writing the answer in the space provided.

Your mother Yes No

28.7 Is your mother still alive ☐ ☐

If No, 28.8 **How old was she when he died?___________years**

28.9 What were you told was the cause of her death. Please tick only one cause.

Heart attack	☐ 1	Other cancer	☐ 6
High blood pressure	☐ 2	Accident or injury	☐ 7
Stroke	☐ 3	Other cause	☐ 8
Respiratory disease	☐ 4	Don't know	☐ 9
Cancer of breast	☐ 5		

Family history of heart attacks and stroke

Are any of your relations affected by heart attacks and strokes either now or before they died?

**Mother**	Yes	No	Don't know
28.10 Heart attack	☐	☐	☐
28.11 Stroke	☐	☐	☐
**Father**			
28.12 Heart attack	☐	☐	☐
28.13 Stroke	☐	☐	☐

**Sisters**	Yes	No	Don't know	No sisters or brothers
	☐	☐	☐	☐

28.14 Heart attack				
28.15 Stroke	☐	☐	☐	☐
**Brothers**				
28.16 Heart attack	☐	☐	☐	☐
28.17 Stroke	☐	☐	☐	☐

THANK YOU FOR COMPLETING THIS QUESTIONNAIRE.

CHECK CAREFULLY THAT YOU HAVE ANSWERED EACH PAGE AND THEN RETURN IT IN THE REPLY PAID ENVELOPE PROVIDED.
