# Supplementary material for: Geographical variation in cardiovascular incidence: results from the British Women's Heart and Health Study
Source: BMC Public Health. 2010 Nov 15;10:696. doi: 10.1186/1471-2458-10-696 (PMC2996371; doi:10.1186/1471-2458-10-696)
Supplement: Additional file 2 — 3-year follow-up questionnaire (long form). A self administered questionnaire about lifestyle and medical history which was sent out in March 2003. [file 1471-2458-10-696-S2.PDF]

BRITISH WOMEN'S HEART & HEALTH STUDY

FOLLOW-UP QUESTIONNAIRE 2003

Thank you for taking part in the British Women's Heart and Health Study. It would be very helpful if you could complete this questionnaire, which will bring us up to date with your health and lifestyle.

Most questions can be answered simply by ticking the appropriate box ☑.
Some questions ask for a date as well, please give this if you can.

All your answers will be treated as **strictly confidential** and will only be seen by the research team.

Please complete the form as soon as possible and return in the reply paid envelope. No stamp is required.

If you would like a copy of this questionnaire in large print, or have any other difficulties with the questions, please ring Rita Patel or Kath Wornell on 0117 9287392 and leave your phone number so we can call you back.

THANK YOU FOR YOUR HELP.

British Women's Heart & Health Study
Department of Social Medicine
University of Bristol
Canynge Hall
Whiteladies Road
Bristol BS8 2PR

Office use only
Identification label here

Contact details

1.1 Your full name:	…………………………………………………………………
1.2 Your maiden name (if applicable):	…………………………………………………………………
1.3 Your address:	…………………………………………………………………<br>…………………………………………………………………<br>…………………………………………………………………<br>…………………………………………………………………
1.4 Your postcode:	………………………………………….
1.5 Your telephone number:	(……………..) …………………………<br>*area code*
1.6 Your date of birth:	….. / ……… / 19……<br>day month year
1.7 Name of your GP:	…………………………………………………………………
1.8 GP Address:	…………………………………………………………………<br>…………………………………………………………………<br>…………………………………………………………………<br>…………………………………………………………………
1.9 GP Postcode:	………………………………………….

Office use only
Identification label here

Your health at present

2.1 Compared with other women of your age, how would you describe your health at present?

Please tick one box only

Excellent	☐ 1
Good	☐ 2
Fair	☐ 3
Poor	☐ 4

Conditions affecting the heart or circulation

Have you ***ever*** been told by a doctor that you have had any of the following conditions? **Please answer each question.**

		(a) Yes 1	(a) No 2	(b) **If yes**, please give year of most recent diagnosis
3.1	Heart attack (coronary thrombosis or myocardial infarction)	☐	☐	______
3.2	Heart failure	☐	☐	______
3.3	Angina	☐	☐	______
3.4	Other heart trouble	☐	☐	______
3.5	Aortic aneurysm	☐	☐	______
3.6	Narrowing or hardening of the arteries in the leg (including claudication)	☐	☐	______
3.7	High blood pressure	☐	☐	______
3.8	High cholesterol	☐	☐	______

Stroke

		(a) Yes 1	(a) No 2	(b) **If yes**, please give year of the most recent stroke
4.1	Have you ***ever*** been told by a doctor that you have had a stroke?	☐	☐	______
	**If yes,**			
4.2	did symptoms last for more than 24 hours?	☐	☐	
4.3	have you made a complete recovery from your stroke?	☐	☐	
4.4	in the last fortnight did you require help from another person in day-to-day activities?	☐	☐	

Investigations and treatment for heart trouble

Have you ever had any of the following **tests or treatment** for chest pain or heart disease?

Please answer each question. If yes, please complete as much information as possible.

		(a) Yes₁	(a) No₂	(b) **If yes,** what year	(c) Where? NHS₁	(c) Where? Private₂
5.1	An exercise ECG (treadmill) test	☐	☐	________	☐	☐
5.2	Angiogram or X-ray of your coronary arteries (a dye of the arteries)	☐	☐	________	☐	☐
5.3	Angioplasty of coronary arteries (balloon treatment for angina)	☐	☐	________	☐	☐
5.4	Coronary artery bypass graft ('CABG' or 'CABBAGE') operation	☐	☐	________	☐	☐
5.5	An admission to hospital with chest pain, angina or heart attack	☐	☐	________	☐	☐
5.6	A GP referral to a hospital to see a heart specialist	☐	☐	________	☐	☐
5.7	A GP referral to a chest pain clinic	☐	☐	________	☐	☐
5.8	An echocardiogram or ultrasound on the chest	☐	☐	________	☐	☐
5.9	Other tests, investigations or operations on the heart, arteries or veins<br>If yes, please give details below:	☐	☐	________	☐	☐

____

Cancer

		(a) Yes₁	(a) No₂	(b) **If yes**, what type of cancer	(c) Year diagnosed
6.1	Have you ***ever*** been told by a doctor that you have cancer?	☐	☐	________	________
6.2				________	________
6.3				________	________

Conditions of joints and bones

		(a) Yes₁	(a) No₂	(b) **If yes**, year first diagnosed
7.1	Have you ***ever*** been told by a doctor that you have arthritis?	☐	☐	________
7.2	Have you ***ever*** been told by a doctor that you have osteoporosis?	☐	☐	________

Respiratory problems

		(a) Yes₁	No₂	(b) **If yes**, year first diagnosed
8.1	Have you ***ever*** been told by a doctor that you have chronic bronchitis or emphysema?	☐	☐	________
8.2	Have you ***ever*** been told by a doctor that you have asthma?	☐	☐	________

Diabetes

Do any of the following family members have **diabetes**? *(Please include those who have died)*

		(a) Yes₁	No₂	(b) Age at which diabetes started
9.1	Mother	☐	☐	________
9.2	Father	☐	☐	________
9.3	Sister (1)	☐	☐	________
9.4	Sister (2)	☐	☐	________
9.5	Sister (3)	☐	☐	________
9.6	Brother (1)	☐	☐	________
9.7	Brother (2)	☐	☐	________
9.8	Brother (3)	☐	☐	________

		(a) Yes₁	No₂	(b) **If yes**, year first diagnosed
9.9	Have you ***ever*** been told by a doctor that you have diabetes?	☐	☐	________

Urinary incontinence

		Yes₁	No₂
10.1	During the last 12 months have you lost urine involuntarily / accidentally? Please answer yes for any amount of urine.	☐	☐
10.2	**If yes,** have you been to a doctor with this problem?	☐	☐

		(a) Yes₁	No₂	(b) **If yes**, year first diagnosed
10.3	Have you ***ever*** been told by a doctor that you have **stress** incontinence?	☐	☐	________
10.4	Have you ***ever*** been told by a doctor that you have **urge** incontinence?	☐	☐	________

Seeing

		Yes 1	No 2
11.1	Can you see well enough to recognise a friend at a distance of four yards (across a room), with glasses if used?	☐	☐
11.2	**If no**, can you see well enough to recognise a friend at a distance of one yard, with glasses if used?	☐	☐

Hearing

		Yes 1	No 2
11.3	Is your hearing good enough to follow a TV programme at a volume others find acceptable?	☐	☐
11.4	**If no,** can you follow a TV programme with the volume turned up?	☐	☐

Operations

		(a) Yes 1	(a) No 2	(b) **If yes**, what type of operation	(c) Year
12.1	Have you had any operations in the last **5 years**?	☐	☐	____________	______
12.2				____________	______
12.3				____________	______

Weight

13.1 What is your present weight (in indoor clothes, without shoes)? ________Stones and______Pounds

or _______ Kilograms

13.2 If possible, please use scales to weigh yourself.
If you have no scales and have made an estimate please tick here ☐

		About right 1	Too high 2	Too low 3
13.3	Do you consider your weight at present to be?	☐	☐	☐

13.4 Has your weight changed in the last 4 years?

- Yes, decreased a lot ☐ 1 **Please tick one box only**
- Yes, decreased a little ☐ 2
- No, not changed ☐ 3
- Yes, increased a little ☐ 4
- Yes, increased a lot ☐ 5

13.5 If your weight has decreased in the last 4 years was this…

- Unintentional ☐ 1 **Please tick one box only**
- Intentional for personal reasons ☐ 2
- Intentional because of doctors advice? ☐ 3

Medications / Treatments

		Yes₁	No₂
14.1	Do you take any medication?	☐	☐

If yes, which medications are you taking? Please list all below.

N.B. Please include prescribed tablets, painkillers, medicines, inhalers, sprays, injections AND medications, vitamins and minerals that you buy yourself.

14.2	**Medication** (a)	**Amount and how often (copy details from container)** (b)	**Reason for taking** (c)
1			
2			
3			
4			
5			
6			
7			
8			
9			
10			
11			
12			

If you need more space please continue on the back of the questionnaire.

Preventive health

Have you *ever* had any of the following:
Please answer each question.

		(a) Yes₁	No₂	(b) **If yes,** year of most recent
15.1	Blood pressure check	☐	☐	________
15.2	Blood cholesterol check	☐	☐	________
15.3	Flu vaccination	☐	☐	________
15.4	Dental check	☐	☐	________
15.5	Eye examination/check	☐	☐	________
15.6	Breast cancer screening	☐	☐	________
15.7	Foot care from a chiropodist?	☐	☐	________

Chest pain

		Yes₁	No₂	
16.1	Do you ever have any pain or discomfort in your chest?	☐	☐	
	If yes, is the chest pain produced when you	Yes₁	No₂	Unable to walk₃
16.2	…walk at an ordinary pace on the level?	☐	☐	☐
16.3	…walk uphill or hurry?	☐	☐	☐

Breathlessness

		Yes₁	No₂	Never do this₃	Unable to walk₄
17.1	Are you troubled by shortness of breath when hurrying on level ground or walking up a slight hill?	☐	☐	☐	☐
17.2	Do you get short of breath walking with other people of your own age on level ground?	☐	☐	☐	☐
17.3	In the past twelve months have you at any time been awoken at night by an attack of shortness of breath?	☐	☐		

Limitations in activities

Do you currently have **difficulty carrying out** any of the following activities?
Please answer each question.

		Yes₁ Difficulty	No₂ Difficulty
18.1	Going up or down stairs……………..	☐	☐
18.2	Bending down……………………….	☐	☐
18.3	Straightening up……………………..	☐	☐
18.4	Keeping your balance……………….	☐	☐
18.5	Going out of the house………………	☐	☐
18.6	Walking 400 yards…………………..	☐	☐

		(a) Yes₁	(a) No₂	(b) **If yes,** how many times
18.7	Have you had a fall in the last 12 months?	☐	☐	________________

Is your present state of health causing problems with any of the following?
Please answer each question.

		Yes[1] Problems	No[2] Problems
18.8	Family relationships…………………	☐	☐
18.9	Household chores……………………	☐	☐
18.10	Social life……………………………	☐	☐
18.11	Sex life………………………………	☐	☐
18.12	Interests and hobbies………………..	☐	☐
18.13	Holidays and outings………………..	☐	☐
18.14	Job (paid or voluntary)………………	☐	☐

Activities of Daily Living

We need to understand difficulties people may have with various activities because of a health, emotional or physical problems. Do you have **any difficulty** with any of the following activities:

Please answer each question.

		Yes, have difficulty[1]	No, have no difficulty[2]	Never do this[3]
19.1	Crossing a road	☐	☐	☐
19.2	Getting up from a chair after sitting for long periods	☐	☐	☐
19.3	Reaching or extending your arms above shoulder level	☐	☐	☐
19.4	Pulling or pushing large objects like a living room chair	☐	☐	☐
19.5	Lifting or carrying weights over 10 pounds, like a heavy bag of groceries	☐	☐	☐
19.6	Threading a needle	☐	☐	☐
19.7	Dressing, including putting on shoes and socks	☐	☐	☐
19.8	Walking across a room	☐	☐	☐
19.9	Bathing or showering	☐	☐	☐
19.10	Eating, including cutting up your food	☐	☐	☐
19.11	Getting in and out of bed	☐	☐	☐
19.12	Using the toilet, including getting up and down	☐	☐	☐
19.13	Preparing a hot meal	☐	☐	☐
19.14	Shopping for groceries	☐	☐	☐
19.15	Making telephone calls	☐	☐	☐
19.16	Taking medications	☐	☐	☐
19.17	Doing work around the house or garden	☐	☐	☐
19.18	Managing money, such as paying bills and keeping track of expenses?	☐	☐	☐

Smoking

		Yes₁	No₂
20.1	Have you ever smoked cigarettes regularly (at least 1 per day)?	☐	☐
20.2	***If yes,*** do you smoke cigarettes at present?	☐	☐

Current Smoking

If you currently smoke:

20.3 How many cigarettes do you smoke a day at present? ____________ per day

		Yes$_1$	No$_2$
20.4	Do you want to give up?	☐	☐

Have you had any of the following to help you cut down your smoking and did any of them help? **Please answer each question.**

		Yes, helped me to cut down$_1$	Yes, made no difference to my smoking$_2$	Not had$_3$
20.5	Advice from doctor or nurse to stop smoking	☐	☐	☐
20.6	Referral to stop smoking clinic	☐	☐	☐
20.7	Nicotine replacement therapy prescribed by a **doctor or nurse** (sprays, gums, imitation cigarettes or patches)	☐	☐	☐
20.8	Nicotine replacement therapy bought **yourself** (sprays, gums, imitation cigarettes or patches)	☐	☐	☐
20.9	Medication (e.g. Zyban)	☐	☐	☐
20.10	Acupuncture	☐	☐	☐
20.11	Hypnosis	☐	☐	☐
20.12	Advice from family or friends	☐	☐	☐
20.13	Other **If yes**, please describe below	☐	☐	☐

____

Ex Smoking

If you have smoked in the past but have given up:

20.14 When did you stop smoking? Year ____________________

Did you have any of the following to help you stop smoking and did they help you stop?
Please answer each question.

		Yes, helped me to cut down[1]	Yes, made no difference to my smoking[2]	Not had[3]
20.15	Advice from doctor or nurse to stop smoking	☐	☐	☐
20.16	Referral to stop smoking clinic	☐	☐	☐
20.17	Nicotine replacement therapy prescribed by a **doctor or nurse** (sprays, gums, imitation cigarettes or patches)	☐	☐	☐
20.18	Nicotine replacement therapy bought **yourself** (sprays, gums, imitation cigarettes or patches)	☐	☐	☐
20.19	Medication (e.g. Zyban)	☐	☐	☐
20.20	Acupuncture	☐	☐	☐
20.21	Hypnosis	☐	☐	☐
20.22	Advice from family or friends	☐	☐	☐
20.23	Other **If yes**, please describe below	☐	☐	☐

____

Health Scale

21.1 We have drawn a health scale (rather like a thermometer) on which perfect health is 100 and very poor health is 0. Please put a cross (**X**) on the scale to reflect how good or bad your health is today.

Worst Imaginable Health State — Best Imaginable Health State

0 10 20 30 40 50 60 70 80 90 100

Your health overall

Thinking about your health **today** please tick the **one** statement in **each question block** that is most applicable:

22.1	**Pain/Discomfort**:	I have no pain or discomfort	☐ 1
		I have moderate pain or discomfort	☐ 2
		I have extreme pain or discomfort	☐ 3

22.2	**Usual Activities** (e.g. work, study, housework, family or leisure activities):	I have no problems with performing my usual activities	☐ 1
		I have some problems with performing my usual activities	☐ 2
		I am unable to perform my usual activities	☐ 3

22.3	**Self Care**:	I have no problems with washing and dressing	☐ 1
		I have some problems with washing and dressing	☐ 2
		I am unable to wash and dress myself	☐ 3

22.4	**Mobility**:	I have no problems in walking about	☐ 1
		I have some problems in walking about	☐ 2
		I am confined to a chair/ wheelchair	☐ 3

22.5	**Anxiety / Depression**:	I am not anxious or depressed	☐ 1
		I am moderately anxious and/or depressed	☐ 2
		I am extremely anxious and/or depressed	☐ 3

22.6 On average, how many hours sleep do you have each night? ________________ hours

Your present circumstances

23.1	At present, do you live…	Alone	☐ 1
		With husband or partner	☐ 2
		With other family members	☐ 3
		With other people?	☐ 4

23.2 Are you…

Single, *that is never married*	☐ 1	**Please tick one box only**
Married, first and ONLY marriage	☐ 2	
Remarried, second or subsequent marriage	☐ 3	
Divorced	☐ 4	
Separated	☐ 5	
Widowed	☐ 6	
Other	☐ 7	

23.3 If you are divorced / separated / widowed please give the date: Year

(a) Divorced / separated ______________

(b) Widowed ______________

23.4 Think about when you were in your 40's and looking forward to what your life would be like in the future. Would you say that your life is now…

Better than I expected	☐ 1	**Please tick one box only**
Same as I expected	☐ 2	
Worse than I expected?	☐ 3	

23.5 At what age do you consider someone to be old? ______________ Years

23.6 Do you feel younger, older, or about the same as your actual age?

Younger	☐ 1	**Please tick one box only**
Older	☐ 2	
About the same	☐ 3	

23.7 If you feel younger or older, about what age do you feel? ______________ Years

<u>Your family</u>

How many brothers and sisters do you have? *Please include brothers & sisters who have died*

24.1 Brothers ______________

24.2 Sisters ______________

24.3 Among your brothers and sisters, where do you come in order of birth? ______________

Do any of the following run in your family?
Please answer each question.

		Yes 1	No 2
24.4	Heart attacks	☐	☐
24.5	Strokes	☐	☐
24.6	Cancers	☐	☐
24.7	Diabetes	☐	☐

24.8 If you have children, what age were you when you had your **first** child? Age ________________

If you have children, how tall was the **father** of your **first** child?

Please give actual height if known or else please tick which category applies.

24.9 Height ________feet and_______inches

24.10 **Or** Less than 5 foot ☐ 1

Between 5’1” and 5’6” ☐ 2

Between 5’7” and 6’ ☐ 3

Between 6’1” and 6’6” ☐ 4

Greater than 6’6” ☐ 5

Don’t know? ☐ 6

Your birth weight

What was your birth weight?

Please give actual weight if known or else please tick which category applies.

25.1 Weight ________lbs and_______oz

25.2 **Or** Less than 5lb 8oz ☐ 1

Between 5lb 8oz and 6lb 15oz ☐ 2

Between 7lb and 8lb 15 oz ☐ 3

Between 9lb and 10lb 15oz ☐ 4

Greater than 11lb ☐ 5

Don’t know? ☐ 6

Spending time abroad

		Yes 1	No 2
26.1	Do you own property abroad?	☐	☐

26.2 **If yes,** in which country? ______________________________

		Yes 1	No 2
26.3	Do you currently, or are you planning in the next few years to spend a long period of time (1 month or more) living abroad?	☐	☐

26.4 **If yes,** in which country? ______________________________

Consent

A very important part of this study is to observe the future health of the people taking part. To do this properly, we will need to send questionnaires to you in the future. We also need to obtain routine information about your health and medical care from several national agencies closely related to the National Health Service* and from your medical records, particularly for conditions of the heart and circulation, diabetes, cancer and other disabling conditions and medical problems. You may not have any of these conditions, but that information is just as important for us.

We are therefore seeking your permission to allow us to do this.

Do you agree to allow us to follow your future health through medical records and obtain routine information from the agencies related to the National Health Service*?

☐ Yes, Agreed ☐ No, Not agreed

*these include the National Health Service Central Register (England and Wales), the General Register Office (Scotland), the National Cancer Intelligence Centre, the National Breast Cancer Screening Programme, and the Primary Care Patient Registration Services.

The information included in this questionnaire and obtained from the other sources described above will be stored and processed by the British Women's Heart and Health Study team / University of Bristol. This information will be held and processed **only for the purposes of medical research** and your consent is conditional upon the university complying with its duties and obligations under the Data Protection Act.

Do you agree to the British Women's Heart and Health Study team / University of Bristol recording and processing information about you?

☐ Yes, Agreed ☐ No, Not agreed

Please sign below:

____ Date: ____________________

All the information will be treated **in absolute confidence** by the Research Team.

Please go back and check you have answered all the questions.

Thank you very much for completing the questionnaire.

Please return it to us in the envelope provided. No stamp is needed.

Thank you very much for taking the time to fill in this questionnaire. It is only with your help that we can continue with this valuable research.
