# Supplementary material for: Geographical variation in cardiovascular incidence: results from the British Women's Heart and Health Study
Source: BMC Public Health. 2010 Nov 15;10:696. doi: 10.1186/1471-2458-10-696 (PMC2996371; doi:10.1186/1471-2458-10-696)
Supplement: Additional file 3 — 3-year follow-up questionnaire (short form). A self administered questionnaire about lifestyle and medical history which was sent out in March 2003. [file 1471-2458-10-696-S3.PDF]

British Women's Heart & Health Study
Department of Social Medicine
University of Bristol
Canynge Hall
Whiteladies Road
Bristol BS8 2PR

Dear

Thank you for taking part in the British Women's Heart and Health Study. It would be very helpful if you could complete this questionnaire, which will bring us up to date with your health and lifestyle. All your answers will be treated as **strictly confidential** and will only be seen by our small research team.

Most questions can be answered simply by ticking the appropriate box ☑.
Some questions ask for a date as well, please give this if you can.

Thank you very much.

Your contact details

1.1 Your full name:	………………………………………………………………
1.2 Your maiden name (if applicable):	………………………………………………………………
1.3 Your address:	………………………………………………………………<br>………………………………………………………………<br>………………………………………………………………
1.4 Your postcode:	…………………………………………
1.5 Your telephone number:	(……………..) …………………………<br>*area code*
1.6 Your date of birth:	….. / ……… / 19……<br>day month year
1.7 Name of your GP:	………………………………………………………………
1.8 GP Address:	………………………………………………………………<br>………………………………………………………………<br>………………………………………………………………
1.9 GP Postcode:	…………………………………………

Office use only

Consent

Our research depends on linking information in your medical records and to do this we need your permission. In order to update your health record effectively, we need to obtain routine information from your family doctor and, where appropriate, from hospitals and several National Health Service agencies listed below*. We are particularly concerned to know about illnesses of the heart and circulation, diabetes, cancer and other disabling conditions. Even if you do not have any of these conditions, the review of your medical records is of great importance to us. Your information is kept securely and without personal identifying factors (such as your name). Your information is only used by members of our small research team.

Your consent is entirely voluntary and can be withdrawn at any time. Your medical care from the National Heath Service will not be affected, whether or not you agree to our request.

Do you agree to allow us to follow your future health in this way?

Agreed ☐ **Not agreed** ☐

*The agencies related to the National Health Service are:
- -the National Health Service Central Register (England and Wales),
- -the General Register Office (Scotland),
- -the National Cancer Intelligence Centre,
- -the National Breast Cancer Screening Programme, and
- -the Primary Care Patient Registration Services.

The information included in the questionnaire and obtained from the other sources will be stored and processed by the British Women's Heart and Health Study team / University of Bristol. This information will be held and processed **only for the purposes of medical research**. The university has to comply with its duties and obligations under the Data Protection Act. Your information is kept securely and without personal identifying factors (such as your name). Your information is only used by members of our small research team.

Do you agree to allow us to store the information you have provided in this way?

Agreed ☐ **Not agreed** ☐

Please sign and date below

____ Date: _____ /_____ / 20_____

Office use only

Short questionnaire

Your health at present

2.1 Compared with other women of your age, how would you describe your health at present?

Please tick one box only

Excellent	☐ 1
Good	☐ 2
Fair	☐ 3
Poor	☐ 4

Conditions affecting the heart or circulation

Have you ***ever*** been told by a doctor that you have had any of the following conditions? **Please answer each question.**

		(a) Yes 1	No 2	(b) **If yes**, please give year of most recent diagnosis
3.1	Heart attack (coronary thrombosis or myocardial infarction)	☐	☐	________
3.2	Heart failure	☐	☐	________
3.3	Angina	☐	☐	________
3.4	Other heart trouble	☐	☐	________
3.5	Aortic aneurysm	☐	☐	________
3.6	Narrowing or hardening of the arteries in the leg (including claudication)	☐	☐	________
3.7	High blood pressure	☐	☐	________
3.8	High cholesterol	☐	☐	________

Stroke

		(a) Yes 1	No 2	(b) **If yes**, please give year of the most recent stroke
4.1	Have you ***ever*** been told by a doctor that you have had a stroke?	☐	☐	________
	**If yes,**			
4.2	did symptoms last for more than 24 hours?	☐	☐	
4.3	have you made a complete recovery from your stroke?	☐	☐	
4.4	in the last fortnight did you require help from another person in day-to-day activities?	☐	☐	

Medications / Treatments

		Yes[1]	No[2]
14.1	Do you take any medication?	☐	☐

If yes, which medications are you taking? Please list all below.

N.B. Please include prescribed tablets, painkillers, medicines, inhalers, sprays, injections AND medications, vitamins and minerals that you buy yourself.

14.2	**Medication** (a)	**Amount and how often (copy details from container)** (b)	**Reason for taking** (c)
1			
2			
3			
4			
5			

If you need more space please continue on a separate sheet of paper.

Chest pain

		Yes[1]	No[2]	
16.1	Do you ever have any pain or discomfort in your chest?	☐	☐	
	If yes, is the chest pain produced when you	Yes[1]	No[2]	Unable to walk[3]
16.2	…walk at an ordinary pace on the level?	☐	☐	☐
16.3	…walk uphill or hurry?	☐	☐	☐

Limitations in activities

Do you currently have **difficulty carrying out** any of the following activities?
Please answer each question.

		Yes[1] Difficulty	No[2] Difficulty
18.1	Going up or down stairs……………..	☐	☐
18.2	Bending down……………………….	☐	☐
18.3	Straightening up……………………..	☐	☐
18.4	Keeping your balance……………….	☐	☐
18.5	Going out of the house………………	☐	☐
18.6	Walking 400 yards…………………..	☐	☐

		(a) Yes[1]	(a) No[2]	(b) **If yes,** how many times
18.7	Have you had a fall in the last 12 months?	☐	☐	________

Thank you very much for completing the questionnaire.

Please return it to us in the envelope provided. No stamp is needed.
