# Supplementary material for: Geographical variation in cardiovascular incidence: results from the British Women's Heart and Health Study
Source: BMC Public Health. 2010 Nov 15;10:696. doi: 10.1186/1471-2458-10-696 (PMC2996371; doi:10.1186/1471-2458-10-696)
Supplement: Additional file 4 — 7-year follow-up questionnaire. A third self-administered postal questionnaire was sent in 2007. This was about health and lifestyle to see what was changed, and what has stayed the same since the last survey in 2003. [file 1471-2458-10-696-S4.PDF]

British Women's Heart & Health Study

BRITISH WOMEN'S HEART AND HEALTH STUDY

FOLLOW-UP QUESTIONNAIRE 2007

Thank you for taking part in the British Women's Heart and Health Study. It would be very helpful if you could complete this questionnaire, which tells us about your health and lifestyle.

Most questions can be answered by simply ticking the appropriate box ☑.
Some questions ask for a date as well, please give this if you can.

All your answers will be strictly confidential, and will only be seen by the research team.

Please complete the form as soon as possible, and return it to us in the reply paid envelope. No stamp is required.

If you would like a copy of this questionnaire in large print, or have any other difficulties with the questions, please ring Claire Carson or Antoinette Amuzu on 0207 927 2085.

If you are unable to complete the questionnaire yourself but would like to remain in the study we would be happy for you to ask someone to help you complete the questionnaire. If you have asked someone to help you complete this questionnaire please tick here: ☐ 1
Please tell us the relationship of this person to you:________________________________

THANK YOU FOR YOUR HELP

British Women's Heart and Health Study
Non-Communicable Diseases Epidemiology Unit
London School of Hygiene and Tropical Medicine
Keppel Street
London
WC1E 7HT

Office Use only

Identification label

**Your Contact Details**	
1.1 Your full name:	………………………………………………………………..
1.2 Your maiden name (if applicable):	………………………………………………………………..
1.3 Your address:	…………………………………………………………………<br>…………………………………………………………………<br>…………………………………………………………………<br>…………………………………………………………………
1.4 Your postcode	………………….……………………………………………..
1.5 Your telephone number	(…………………)……………………………………………<br>*area code*
1.6 Your date of birth	……../……./19……<br>*day/month/year*

**Your GP**	
1.7 Name of your GP:	……………………………………………………………...
1.8 GP address:	…………………………………………………………………<br>…………………………………………………………………<br>………………………………………………………………….
1.9 GP postcode:	…………………………………………………………………

**A Contact Person for you**	
If we are unable to reach you, we would like permission to speak to someone else who may be able to tell us where you are. We will only contact this person if we cannot contact you directly.	
1.10 Name of contact person:	………………………………………………………………………….<br>*(title, forename, surname)*
1.11 Relationship(friend/child etc):	………………………………………………………………………
1.12 Address:	………………………………………………………………………<br>………………………………………………………………………
1.13 Telephone number:	(………………..)………………………………………………….

Office Use only

Identification label

Your health at present

2.1 Compared with other women your age, how would you describe your health at present?

Please tick one

Excellent	☐ 1
Good	☐ 2
Fair	☐ 3
Poor	☐ 4

Conditions affecting the heart or circulation

Have you ***ever*** been told that you have had any of the following conditions?
Please answer each question, using a tick

		(a) Yes	(a) No	(b) **If yes**, please give the year of most recent diagnosis
3.1	Heart attack (coronary thrombosis or myocardial infarction)	☐ 1	☐ 2	________
3.2	Heart failure	☐ 1	☐ 2	________
3.3	Angina	☐ 1	☐ 2	________
3.4	Other heart trouble	☐ 1	☐ 2	________
3.5	Aortic aneurysm	☐ 1	☐ 2	________
3.6	Narrowing or hardening of the arteries in the leg (including claudication)	☐ 1	☐ 2	________
3.7	High blood pressure	☐ 1	☐ 2	________
3.8	High cholesterol	☐ 1	☐ 2	________
3.9	Pulmonary Embolism (PE) (blood clot in lung)	☐ 1	☐ 2	________
3.10	Deep Vein Thrombosis (DVT) (blood clot in leg)	☐ 1	☐ 2	________

Stroke

		(a) Yes	(a) No	(b) **If yes,** please give year of most recent stroke
4.1	Have you ***ever*** been told by the doctor that you have had a stroke?	☐ 1	☐ 2	________
	**If Yes,**			
4.2	Did the symptoms last more than 24 hours?	☐ 1	☐ 2	
4.3	Have you made a complete recovery from your stroke?	☐ 1	☐ 2	
4.4	In the last fortnight did you require help from another person in day-to-day activities?	☐ 1	☐ 2	

Investigations and treatment for heart trouble

Have you ever had any of the **following tests or treatment** for chest pain or heart disease?

Please answer each question. If yes, please complete as much information as possible

		(a) Yes	(a) No	(b) If yes, what year	(c) Where? NHS	(c) Where? Private
5.1	An exercise ECG (treadmill) test	☐ 1	☐ 2		☐ 1	☐ 2
5.2	Angiogram or x-ray of your coronary arteries (a dye of the arteries)	☐ 1	☐ 2		☐ 1	☐ 2
5.3	Angioplasty of the coronary arteries (balloon treatment for angina)	☐ 1	☐ 2		☐ 1	☐ 2
5.4	Coronary artery bypass graft ('CABG' or 'CABBAGE') operation	☐ 1	☐ 2		☐ 1	☐ 2
5.5	An admission to hospital with chest pain, angina or heart attack	☐ 1	☐ 2		☐ 1	☐ 2
5.6	A GP referral to a hospital to see a heart specialist	☐ 1	☐ 2		☐ 1	☐ 2
5.7	A GP referral to a chest pain clinic	☐ 1	☐ 2		☐ 1	☐ 2
5.8	An echocardiogram or ultrasound of the chest	☐ 1	☐ 2		☐ 1	☐ 2
5.9	Other tests, investigations or operations on the heart, arteries or veins<br>If yes, please give details below:					
5.10						
5.11						

Cancer

		(a) Yes	(a) No	(b) **If yes,** what type of cancer?	(c) Year diagnosed?
6.1	Have you ***ever*** been told by a doctor that you have cancer?	☐ 1	☐ 2		
6.2	*Please list any other types of cancer*				
6.3					
6.4					

Conditions of the joints and bones

		(a) Yes	No	(b) Year diagnosed?
7.1	Have you ever been told by a doctor that you have osteoporosis?	☐ 1	☐ 2	________
7.2	Have you ever been told by a doctor that you have arthritis?	☐ 1	☐ 2	________

7.3 **If yes** please give the type of arthritis if known:

Osteoarthritis ☐ 1
Rheumatoid arthritis ☐ 2
Other (please give details) ☐ 3

____

____

Which joints are affected? *(please tick all that apply)*

7.4	Knees	☐ 1	7.7	Back	☐ 1
7.5	Hips	☐ 1	7.8	Neck	☐ 1
7.6	Hands and / or wrists	☐ 1	7.9	Shoulders	☐ 1

7.10 Other (please give details): ________

Falls and fractures

		Yes	No	
8.1	Have you had a fall in the last 12 months?	☐ 1	☐ 2	**If yes**, go to 8.2<br>**If no**, go to 8.4
8.2	**If yes:** How many times have you fallen?	________		times
8.3	Did you seek medical attention?	☐ 1	☐ 2	

		(a) Yes	No	(b) Year of last fracture
8.4	Have you ever fractured your hip?	☐ 1	☐ 2	________
8.5	Have you ever fractured your wrist?	☐ 1	☐ 2	________

Respiratory problems and breathlessness

		Yes	No	Never do this	Unable to walk
9.1	Are you troubled by shortness of breath when hurrying on level ground or walking up a slight hill?	☐ 1	☐ 2	☐ 3	☐ 4
9.2	Do you get short of breath walking with other people of your own age on level ground?	☐ 1	☐ 2	☐ 3	☐ 4
9.3	In the past twelve months have you at any time been awoken at night by an attack of shortness of breath?	☐ 1	☐ 2		
9.4	Have you ever been told by a doctor that you have chronic bronchitis or emphysema?	☐ 1	☐ 2		
9.5	Have you ever been told by a doctor that you have asthma?	☐ 1	☐ 2		

Chest pain

		Yes	No	Never do this	Unable to walk
10.1	Do you ever have any pain or discomfort in your chest?	☐ 1	☐ 2		
	If yes, is this chest pain produced when you…				
10.2	… walk at an ordinary pace on level ground?	☐ 1	☐ 2	☐ 3	☐ 4
10.3	… walk uphill or hurry?	☐ 1	☐ 2	☐ 3	☐ 4

Diabetes

		Yes	No	
11.1	Have you ever been told that you have diabetes?	☐ 1	☐ 2	**If yes**, go to 11.2<br>**If no,** go to 12.1
11.2	**If yes:** What year was this first diagnosed?	________________		

			Yes	No
11.3	How is your Diabetes controlled?	Diet	☐ 1	☐ 2
11.4		Tablets	☐ 1	☐ 2
11.5		Insulin	☐ 1	☐ 2

Sight and Hearing

		Yes	No	
12.1	Can you see well enough to recognise a friend across a room, with glasses if used?	☐ 1	☐ 2	**If yes,** go to 12.3<br>**If no,** go to 12.2
12.2	**If no,** can you see well enough to recognise a friend across a table, with glasses if used?	☐ 1	☐ 2	
12.3	Is your hearing good enough to follow a TV programme at a volume others find acceptable, with a hearing aid if necessary?	☐ 1	☐ 2	**If yes,** go to 12.5<br>**If no,** go to 12.4
12.4	**If no,** can you follow a TV programme with the volume turned up, with a hearing aid if necessary?	☐ 1	☐ 2	

12.5 **If you own a hearing aid,** how often do you wear it?

☐ 1 I rarely wear it ☐ 2 I wear it most days ☐ 3 I always wear my hearing aid

Operations

		(a) Yes	No	(b) **If yes**, what type of operation?	(c) Year
13.1	Have you had any operations in the last **4 years?**	☐ 1	☐ 2	____________________	__________
13.2	*Please list all operations*			____________________	__________
13.3	*If you need more space please*			____________________	__________
13.4	*continue on another sheet of*			____________________	__________
13.5	*paper*			____________________	__________

Weight and Waist measurement

14.1 What is your present weight (in indoor clothes, without shoes)? _________ st ________ lbs

OR __________ kilograms

14.2 If possible, please use scales to weigh yourself. If you have no scales and have made an estimate please tick this box: ☐ 1

14.3 Do you consider your present weight to be…

☐ 1 About right
☐ 2 Too high
☐ 3 Too low

14.4 Has your weight changed in the last 4 years?

☐ 1 Yes, decreased a lot
☐ 2 Yes, decreased a little
☐ 3 No, not changed
☐ 4 Yes, increased a little
☐ 5 Yes, increased a lot

14.5 If your weight has decreased in the last 4 years was this…

☐ 1 Unintentional
☐ 2 Intentional, for personal reasons
☐ 3 Intentional, because of doctor's advice
☐ 4 My weight has not changed

We would also like you to **measure your waist**. We have included a **tape measure and instructions** describing how we want you to take and record your waist measurement. Please read these, and remember to include the tape measure when you return this questionnaire to us. **(If you did not receive a tape measure please call the study team on 0207 927 2085)**.

14.6 After you have measured your waist, please write the results here: _________________ inches

Preventative care

Have you had any of the following in the **last 4 years**?
Please answer each question

		(a) Yes	(a) No	(b) **If yes**, year of most recent check
15.1	Blood pressure check	☐ 1	☐ 2	______________
15.2	Blood cholesterol check	☐ 1	☐ 2	______________
15.3	Flu vaccination	☐ 1	☐ 2	______________
15.4	Dental check	☐ 1	☐ 2	______________
15.5	Eye examination / check	☐ 1	☐ 2	______________
15.6	Breast cancer screening	☐ 1	☐ 2	______________
15.7	Foot care from a chiropodist	☐ 1	☐ 2	______________

Medications and Treatments

16.1 Do you take any regular medication? Yes ☐ 1 No ☐ 2 **If no**, go to 17.1

16.2 **If yes**, which medication are you taking?

N.B. Please include prescribed tablets, painkillers, medicines, inhalers, sprays, injections AND medications, vitamins and minerals that you buy yourself

	**Name of Medication** (Please copy name in full from container) **(a)**	**Amount, and how often** (please copy from container) **(b)**	**Reason for taking (c)**
1			
2			
3			
4			
5			
6			
7			
8			
9			
10			
11			
12			
13			
14			
15			

If you need more space, please continue on a separate sheet of paper

The Health Scale

17.1 We have drawn a health scale (rather like a thermometer) on which perfect health is 100 and poor health is 0. Please put an (**X**) on the scale to reflect how good or bad your health is **today**.

Worst imaginable health state

Best imaginable health state

0 10 20 30 40 50 60 70 80 90 100

Your Overall Health Today

By placing a tick in ONE box in EACH group below, please indicate which statement best describes your own health state today.

Please tick ONE per question

18.1 **Pain / Discomfort**

- I have no pain or discomfort ☐ 1
- I have moderate pain or discomfort ☐ 2
- I have extreme pain or discomfort ☐ 3

18.2 **Usual Activities**

- I have no problems with performing my usual activities ☐ 1
- I have some problems with performing my usual activities ☐ 2
- I am unable to perform my usual activities ☐ 3

18.3 **Self Care**

- I have no problems with washing and dressing ☐ 1
- I have some problems with washing and dressing ☐ 2
- I am unable to wash and dress myself ☐ 3

18.4 **Mobility**

- I have no problems in walking about ☐ 1
- I have some problems in walking about ☐ 2
- I am confined to a chair / wheelchair ☐ 3

18.5 **Anxiety / Depression**

- I am not anxious or depressed ☐ 1
- I am moderately anxious and / or depressed ☐ 2
- I am extremely anxious and / or depressed ☐ 3

About your quality of life

By placing a tick in ONE box in EACH group below, please indicate which statement best describes your quality of life at the moment.

Please tick ONE per question

19.1 **Love and Friendship**

- I have **all** of the love and friendship that I want ☐ 1
- I can have **a lot** of the love and friendship that I want ☐ 2
- I can have a **little** of the love and friendship that I want ☐ 3
- I cannot have **any** of the love and friendship that I want ☐ 4

19.2 **Thinking about the future**

- I can think about the future **without any** concern ☐ 1
- I can think about the future **with only a little** concern ☐ 2
- I can think about the future **with some** concern ☐ 3
- I can only think about the future **with a lot** of concern ☐ 4

19.3 **Doing things that make you feel valued**

I am able to do **all** of the things that make me feel valued	☐	1
I am able to do **many** of the things that make me feel valued	☐	2
I am able to do **a few** of the things that make me feel valued	☐	3
I am unable to do **any** of the things that make me feel valued	☐	4

19.4 **Enjoyment and pleasure**

I can have **all** of the pleasure and enjoyment that I want	☐	1
I can have **a lot** of the pleasure and enjoyment that I want	☐	2
I can have **a little** of the pleasure and enjoyment that I want	☐	3
I cannot have **any** of the pleasure and enjoyment that I want	☐	4

19.5 **Independence**

I am able to be **completely** independent	☐	1
I am able to be independent in **many things**	☐	2
I am able to be independent in **a few things**	☐	3
I am **unable** to be at all independent	☐	4

Limitations in activities (please answer each question)

Do you currently have difficulty carrying out any of the following activities?

		**Yes,** I have difficulty	**No**, I have no difficulty
20.1	Going up or down stairs	☐ 1	☐ 2
20.2	Bending down	☐ 1	☐ 2
20.3	Straightening up	☐ 1	☐ 2
20.4	Keeping your balance	☐ 1	☐ 2
20.5	Going out of the house	☐ 1	☐ 2
20.6	Walking 400 yards	☐ 1	☐ 2

Is your present state of health causing problems with any of the following?

		**Yes**, it is causing problems	**No**, it is not causing problems
20.7	Family relationships	☐ 1	☐ 2
20.8	Household chores	☐ 1	☐ 2
20.9	Social life	☐ 1	☐ 2
20.10	Sex life	☐ 1	☐ 2
20.11	Interests and hobbies	☐ 1	☐ 2
20.12	Holidays and outings	☐ 1	☐ 2
20.13	Job (paid or voluntary)	☐ 1	☐ 2

Activities of daily life

We need to understand difficulties that people may have with various activities because of their health, emotional or physical problems. Do you have any difficulty with any of the following activities

		**Yes,** I have difficulty/need help	**No**, I have no difficulty	I **never** do this
21.1	Using public transport on your own	☐ 1	☐ 2	☐ 3
21.2	Driving a car on your own	☐ 1	☐ 2	☐ 3
21.3	Crossing a road	☐ 1	☐ 2	☐ 3
21.4	Getting up from a chair after sitting for a long period	☐ 1	☐ 2	☐ 3
21.5	Reaching or extending your arms above shoulder level	☐ 1	☐ 2	☐ 3
21.6	Pulling or pushing large objects like a living room chair	☐ 1	☐ 2	☐ 3
21.7	Lifting or carrying weights over 10 pounds, like a heavy bag of groceries	☐ 1	☐ 2	☐ 3
21.8	Gripping with your hands, such as opening a jam jar	☐ 1	☐ 2	☐ 3
21.9	Threading a needle	☐ 1	☐ 2	☐ 3
21.10	Cutting your toe nails	☐ 1	☐ 2	☐ 3
21.11	Dressing, including putting on shoes and socks	☐ 1	☐ 2	☐ 3
21.12	Walking across a room	☐ 1	☐ 2	☐ 3
21.13	Bathing or showering	☐ 1	☐ 2	☐ 3
21.14	Eating, including cutting up your food	☐ 1	☐ 2	☐ 3
21.15	Getting in and out of bed	☐ 1	☐ 2	☐ 3
21.16	Using the toilet, including getting up and down	☐ 1	☐ 2	☐ 3
21.17	Preparing a hot meal	☐ 1	☐ 2	☐ 3
21.18	Shopping for groceries	☐ 1	☐ 2	☐ 3
21.19	Making telephone calls by yourself	☐ 1	☐ 2	☐ 3
21.20	Taking medications by yourself	☐ 1	☐ 2	☐ 3
21.21	Doing light housework, such as washing up	☐ 1	☐ 2	☐ 3
21.22	Doing work around the house or garden	☐ 1	☐ 2	☐ 3
21.23	Managing money, paying bills or keeping track of expenses	☐ 1	☐ 2	☐ 3

Sleep

22.1 On average, how many hours sleep do you have each night? ______________ hours

Your bladder

Many people leak urine some of the time. We are trying to find out how many people leak urine, and how much it bothers them. We would be grateful if you could answer the following questions, thinking about your symptoms in **the last month**.

23.1 Does urine leak before you can get to a toilet?

- ☐ 1 Never
- ☐ 2 Occasionally
- ☐ 3 Sometimes
- ☐ 4 Most of the time
- ☐ 5 All of the time

23.2 How often do you leak urine?

- ☐ 1 Never
- ☐ 2 Once or less per week
- ☐ 3 2-3 times per week
- ☐ 4 Once per day
- ☐ 5 Several times per day

23.3 Does urine leak when you are physically active, exert yourself, cough or sneeze?

- ☐ 1 Never
- ☐ 2 Occasionally
- ☐ 3 Sometimes
- ☐ 4 Most of the time
- ☐ 5 All of the time

23.4 Do you ever leak for no obvious reason and without feeling that you want to go?

- ☐ 1 Never
- ☐ 2 Occasionally
- ☐ 3 Sometimes
- ☐ 4 Most of the time
- ☐ 5 All of the time

23.5 Do you leak urine when you are asleep?

- ☐ 1 Never
- ☐ 2 Occasionally
- ☐ 3 Sometimes
- ☐ 4 Most of the time
- ☐ 5 All of the time

Diet – Milk

We are interested in some specific parts of your diets. We are not asking about all the foods that you eat. The first questions are about **cow's milk only**

24.1 Do you drink any cow's milk?

☐ 1 Yes **Please go to 24.11**

☐ 2 No **Please go to 24.2**

If no:

24.2 How old were you when you stopped drinking cow's milk? ☐☐ Years

24.3 Why did you stop drinking milk?

☐ 1 I don't like it

☐ 2 It makes me ill

If it makes you ill, what are your symptoms? *Tick all that apply*

24.4 ☐ 1 Nausea or Vomiting

24.5 ☐ 1 Diarrhoea

24.6 ☐ 1 Bloating / gas

24.7 ☐ 1 Headaches

24.8 ☐ 1 Other

24.9 Have you ever been told you suffer from lactose intolerance?

☐ 1 Yes ☐ 2 No ☐ 9 Don't know

24.10 Have you ever been told you suffer from a milk allergy?

☐ 1 Yes ☐ 2 No ☐ 9 Don't know

24.11 Do any of your children have an intolerance or allergy to milk and dairy products?

☐ 1 Yes ☐ 2 No ☐ 9 I have no children

How often do you eat or drink each of the following **cow's** milk products?

		More than once a day 6	Once a day 5	A couple of days a week 4	Once a week 3	Less than once a week 2	Never 1
24.12	Drinking a glass of milk	☐	☐	☐	☐	☐	☐
24.13	Milk in other drinks (e.g. tea or coffee)	☐	☐	☐	☐	☐	☐
24.14	Cheese	☐	☐	☐	☐	☐	☐
24.15	Yoghurt	☐	☐	☐	☐	☐	☐
24.16	Cream	☐	☐	☐	☐	☐	☐
24.17	Ice Cream	☐	☐	☐	☐	☐	☐
24.18	Cows milk in other foods (rice pudding etc)	☐	☐	☐	☐	☐	☐

Diet – Green vegetables

We are also interested in the green vegetables you eat. In particular we want to know about how much broccoli, cauliflower, cabbage, and Brussels sprouts you eat.

How often do you eat or drink each of the following vegetables?

		More than once a day 6	Once a day 5	A couple of days a week 4	Once a week 3	Less than once a week 2	Never 1
25.1	Cabbage	☐	☐	☐	☐	☐	☐
25.2	Cauliflower	☐	☐	☐	☐	☐	☐
25.3	Broccoli	☐	☐	☐	☐	☐	☐
25.4	Brussels sprouts	☐	☐	☐	☐	☐	☐

25.5 How do you **usually** cook your green vegetables? *(tick **one only** please)*

☐ 1 Lightly Boil ☐ 3 Microwave ☐ 5 Raw

☐ 2 Heavily Boil ☐ 4 Stir fry ☐ 6 Steam

If you do not eat these very often, can you tell us why?: *Tick all that apply*

25.6 ☐ 1 I don’t like the texture

25.7 ☐ 1 They make me feel ill

25.8 ☐ 1 I don’t like the taste

25.9 ☐ 1 I don’t like to cook them

25.10 ☐ 1 They are too expensive

25.11 ☐ 1 No-one else will eat them

Physical Activity

26.1 Which of the following forms of transport do you use most often? *Please tick one box only*

☐ 1 Car ☐ 2 Public transport ☐ 3 Cycle ☐ 4 Walk ☐ 5 Not applicable

26.2 Do you make regular journeys everyday or most days either walking or cycling?

☐ 1 No ☐ 2 I walk ☐ 3 I cycle ☐ 4 Both

26.3 Which of the following best describes your walking pace?

☐ 1 Slow ☐ 2 Steady average ☐ 3 Fairly brisk ☐ 4 Fast (at least 4miles/hr)

26.4 *If you cycle regularly,* how long do you spend cycling in an average week? ________ Hours/week

26.5 Do you take physical activity (e.g. running, swimming, dancing, golf, tennis, squash, jogging, bowls)?

☐ 1 No ☐ 2 Occasionally (less than monthly) ☐ 3 Frequently (more than monthly)

If you take part in these physical activities frequently, (once a month or more):
How many times on average do you take part in these activities?

26.6 Summer ________________ Times / month

26.7 Winter ________________ Times / month

In a typical week during the past year, how many hours did you spend each week in the following activities? *Please write 0 if you did not do this activity.*

Activity	No.	Season		
Walking to work, shopping or leisure	26.8	Summer	______	Hours/week
	26.9	Winter	______	Hours/week
Cycling, including to work and leisure	26.10	Summer	______	Hours/week
	26.11	Winter	______	Hours/week
Gardening, light e.g. pruning, watering	26.12	Summer	______	Hours/week
	26.13	Winter	______	Hours/week
Gardening, heavy e.g. digging, mowing	26.14	Summer	______	Hours/week
	26.15	Winter	______	Hours/week
Physical exercise e.g. fitness, aerobics	26.16	Summer	______	Hours/week
	26.17	Winter	______	Hours/week
DIY e.g. on house or car	26.18	Summer	______	Hours/week
	26.19	Winter	______	Hours/week
Household activities, light e.g. cooking, washing up	26.20	Summer	______	Hours/week
	26.21	Winter	______	Hours/week
Household activities, heavy e.g. hoovering, windows	26.22	Summer	______	Hours/week
	26.23	Winter	______	Hours/week

26.24 In a typical week in the last year, did you do any of these activities vigorously enough to cause breathlessness, sweating or a faster heartbeat?

☐1 Yes ☐2 No

26.25 **If yes,** for how many minutes each week did you perform vigorous activity? ______ Mins/week

26.26 In a typical week in the last year, how many flights of stairs did you climb a day? ______ Flights/day

26.27 Compared with your activity level four years ago, are you doing:

☐1 More ☐2 Same ☐3 Less

26.28 If less, please give a reason: ____________________ ☐ office code

26.29 Compared with other women your age, are you:

☐1 Much more active ☐2 More active ☐3 Similar ☐4 Less active ☐5 Much less active

Where you live

The next questions ask about *your* local area. We want to know how you feel about the place that you live, and what it is like to live there.

In your neighbourhood, how much of a problem are the following?

Please tick one box one each line

		Serious problem	Minor problem	Not a problem
27.1	Vandalism	☐1	☐2	☐3
27.2	Litter and rubbish	☐1	☐2	☐3
27.3	Smells and fumes	☐1	☐2	☐3
27.4	Assaults and muggings	☐1	☐2	☐3
27.5	Burglaries	☐1	☐2	☐3
27.6	Disturbance by children or youngsters	☐1	☐2	☐3
27.7	Speeding traffic	☐1	☐2	☐3
27.8	Discarded needles and syringes	☐1	☐2	☐3
27.9	Uneven or dangerous pavements	☐1	☐2	☐3
27.10	Nuisance from dogs	☐1	☐2	☐3
27.11	Reputation of the neighbourhood	☐1	☐2	☐3
27.12	Lack of safe places for children to play	☐1	☐2	☐3
26.13	Lack of recreational facilities	☐1	☐2	☐3

27.14 Can you see greenery (a garden, trees, park or countryside) from your main living space?

☐1 Yes ☐2 No

27.15 Can you do most of your regular shopping (food, household necessities etc) at shops within easy walking distance (less than 15 minutes) of your home?

☐1 Yes ☐2 No ☐3 Someone else shops for me

Present circumstances

28.1 At present do you live…

☐ 1 Alone
☐ 2 With husband or partner
☐ 3 With other family members
☐ 4 With other people? **If yes**, please describe their relationship to you:

____

28.2 Are you… *Please tick one box only*

☐ 1 Single, *i.e. never married*
☐ 2 Married, first and ONLY marriage
☐ 3 Remarried, second or subsequent marriage
☐ 4 Divorced
☐ 5 Separated
☐ 6 Widowed
☐ 7 Other

28.3 If you are divorced / separated / widowed please give the date : Year

(a) Divorced / separated ____________________

(b) Widowed ____________________

Your family – parents and grandparents

We would like to know a bit more about your family history.
When were your parents and grandparents born? And where? If you do not know precisely please give as much detail as you can.

		(a) Year of birth	Place of birth (b) Town or village	(c) County	(d) Country
29.1	Your mother				
29.2	Your father				
29.3	Your grandmother (father's side)				
29.4	Your grandfather (father's side)				
29.5	Your grandmother (mother's side)				
29.6	Your grandfather (mother's side)				

Your family – your children

We would like to know if your children are healthy. We are also interested in how families move around over the years, so we would like to know where your children were born and where they live now. Please continue on the back page if you need more space.

30	(a) Year of birth	(b) Sex (male$_1$ or female$_2$)	(c) Where was this child born? (Town or village, County and Country)	(d) Where does this person live now? (Town or village, County and Country)	(e) Please give the **first 4 digits** of their current postcode:	Has this person had any of the following health problems? (Yes$_1$, No$_2$ or Don't know$_9$) (g) Angina	(h) Heart Attack or Myocardial Infarction	(i) Stroke
e.g.	*1968*	*Male*	*Huddersfield, West Yorkshire, UK*	*Chard, Somerset, UK*	*TA20*	*No*	*No*	*No*
1								
2								
3								
4								
5								

Consent - please complete and sign

Thank you for completing this questionnaire. You completed a consent form at the time of the first survey in 1999-2000. To allow us to continue our work we now need you to complete and sign an updated consent form.

The British Women's Heart and Health Study started as a joint venture between the University of London and the University of Bristol in 1999. The Study Director, Professor Shah Ebrahim, has recently moved to the London School of Hygiene and Tropical Medicine (part of the University of London), so we need to transfer your data to London to run the project and continue this important research. We will carry on working with our colleagues at the University of Bristol. Both universities fulfill their duties under the Data Protection Act.

Please read the statement carefully and **tick the box** to show that you agree. You must tick the box to remain this study.

Consent to store and process your information

All past and new information that you give us will now be stored and processed by the British Women's Heart and Health Study Team at the University of London. Your information will be held and processed **for the purposes of medical research only** and will be treated in **absolute confidence**.

I agree to allow you to store and process information about me: ☐ 1 **please tick**

Please sign and date below:

Signature:____ Date:_______________

If you change your mind in the future or wish to withdraw from the study you are free to do so at any time. If you have any questions about this form or our work, please call Dr Claire Carson (Study Coordinator) on 0207 927 2085.

Thank you for completing the questionnaire.

Please return it to us in the envelope provided. Please check that you have measured your waist and included the tape measure in the return envelope. No stamp is needed.

Thank you for completing the questionnaire.

Please return it to us in the envelope provided. Please check that you have measured your waist and included the tape measure in the return envelope. No stamp is needed.

British Women's Heart & Health Study
